# Hedgehog proteins create a dynamic cholesterol interface

**DOI:** 10.1371/journal.pone.0246814

**Published:** 2021-02-25

**Authors:** Amirhossein Mafi, Rahul Purohit, Erika Vielmas, Alexa R. Lauinger, Brandon Lam, Yu-Shiuan Cheng, Tianyi Zhang, Yiran Huang, Soo-Kyung Kim, William A. Goddard, Alison E. Ondrus

**Affiliations:** Department of Chemistry, Division of Chemistry & Chemical Engineering, California Institute of Technology, Pasadena, California, United States of America; Rijksuniversiteit Groningen, NETHERLANDS

## Abstract

During formation of the Hedgehog (Hh) signaling proteins, cooperative activities of the Hedgehog INTein (Hint) fold and Sterol Recognition Region (SRR) couple autoproteolysis to cholesterol ligation. The cholesteroylated Hh morphogens play essential roles in embryogenesis, tissue regeneration, and tumorigenesis. Despite the centrality of cholesterol in Hh function, the full structure of the Hint-SRR (“Hog”) domain that attaches cholesterol to the last residue of the active Hh morphogen remains enigmatic. In this work, we combine molecular dynamics simulations, photoaffinity crosslinking, and mutagenesis assays to model cholesterolysis intermediates in the human Sonic Hedgehog (hSHH) protein. Our results provide evidence for a hydrophobic Hint-SRR interface that forms a dynamic, non-covalent cholesterol-Hog complex. Using these models, we suggest a unified mechanism by which Hh proteins can recruit, sequester, and orient cholesterol, and offer a molecular basis for the effects of disease-causing hSHH mutations.

## Introduction

The Hedgehog (Hh) proteins are integral to embryonic development in metazoans [1, 2]. As secreted morphogens, they are responsible for neural tube development, left/right axis specification, and digit formation. Mutations in the human Sonic Hedgehog protein (hSHH) are a leading cause holoprosencephaly (HPE), a congenital syndrome that results in symptoms such as cleft palate and cyclopia [3–5]. In adults, Hh signaling is largely silenced, but it preserves critical functions in neurogenesis, gastrointestinal maintenance, and wound healing [6]. Conversely, aberrant activity of Hh isoforms in adult tissue is a leading driver of tumorigenesis in pancreatic [7], colon [8], prostate [9], and other cancers [10]. Understanding the structure and activity of these proteins is of fundamental significance to human development and disease.

**Fig 1:**
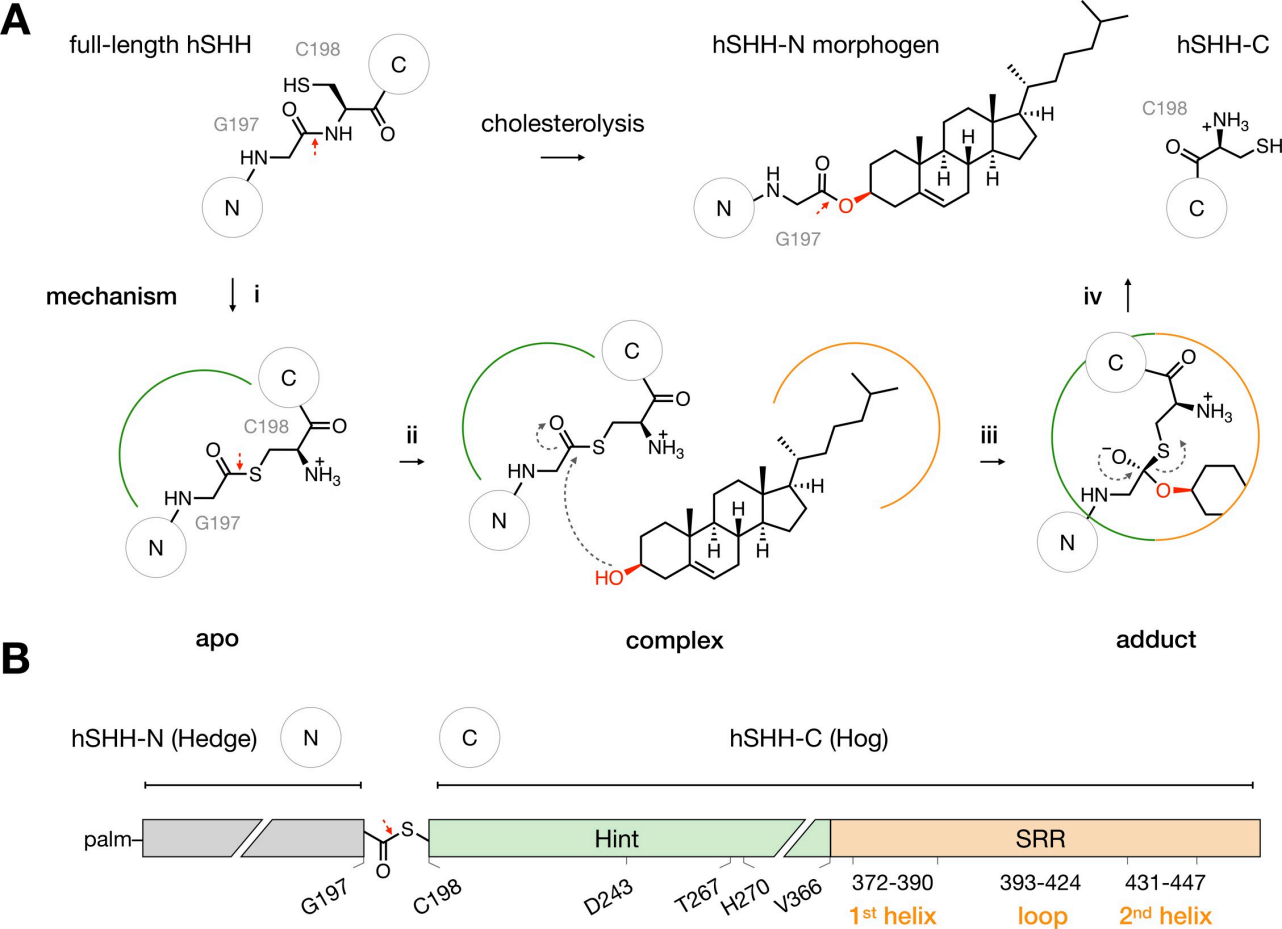
The Hog domain catalyzes Hh-cholesterol ligation. (A) Mechanism of Hh cholesterolysis. (i) In the hSHH protein, the Hint fold catalyzes N-S rearrangement of the amide bond between G197 and C198 to generate a G197-C198 thioester in the hSHH backbone (apo). The original G197-C198 amide bond and the G197-C198 thioester bond are indicated by red arrows. (ii) Cholesterol non-covalently complexes to the Hog domain (complex). (iii) Attack of cholesterol on the G197-C198 thioester forms a covalent hSHH-cholesterol bond (adduct). (iv) Collapse of the adduct releases a cholesterol-esterified hSHH-N morphogen and hSHH-C. (B) Diagram of the hSHH protein. The N-terminal “Hedge” domain (residues C24-G197, grey) functions as the active Hh morphogen after cholesteroylation and N-terminal palmitoylation (palm). The C-terminal “Hog” domain, which facilitates cholesterol adduction, is comprised of the Hint fold (C198-A365, green) and the SRR (V366-S462, orange). Active site residues in the Hint fold and secondary structure motifs in the SRR are indicated.

Hh protein function relies on a self-catalyzed backbone cleavage that requires no external cofactors, accessory proteins, or energy sources [11, 12]. The well-established mechanism of proteolysis occurs *via* intramolecular N-to-S acyl transfer in the protein backbone, templated by a Hedgehog INTein (Hint) fold that can be found in select proteins throughout all kingdoms of life [13, 14]. Hh proteins are unique in that cholesterol intercepts the thioester created by the N-to-S acyl shift, resulting in protein scission and esterification of the last residue of the N-terminal fragment by cholesterol (Fig 1A) [15]. The enabling feature for cholesterol ligation is a C-terminal Sterol Recognition Region (SRR) [12], which together with the Hint domain forms the “Hog” region of the Hh protein (Fig 1B) [16].

Our recent alanine scanning studies have identified residues in the SRR that reduce hSHH cholesterolysis and control the localization of SRR fusion proteins in cells [17], suggesting that the SRR plays roles in both processes. However, the structure of the membrane-associated Hog (Hint-SRR) structure that catalyzes cholesterolysis remains enigmatic. In part, this is due to the biochemical properties of the hSHH Hog domain; its membrane affinity, susceptibility to aggregation, undesired proteolysis, and small size have prevented analysis by x-ray crystallography or high-resolution electron microscopy. More significantly, the dynamic interactions between cholesterol and the Hog protein elude classical methods of structure determination. In this case, chemical tools and molecular dynamics (MD) simulations offer a unique opportunity to study atomic details of these proteins as cohesive structural models [18].

In this study, we use molecular dynamics simulations and experimental data to propose a unified, dynamic Hog model in a cholesterol-containing phospholipid bilayer. Using a cholesterol photoaffinity probe, we capture non-covalent cholesterol-Hog interactions that suggest a hitherto unappreciated interface between the Hint fold and the SRR. Mutations at residues encompassing this interface reinforce potential contacts that localize membrane cholesterol, stabilize a cholesterol-Hog complex, and orient cholesterol for covalent adduction in our models. Our experiments suggest a biophysical basis for mutations in human congenital disorders and expand our understanding of natural small molecule-protein interfaces.

## Materials and methods

### Synthesis of 25-diazirinyl-27-norcholest-5-ene-3β-ol (photocholesterol)

A solution of 27-nor-25-ketocholesterol (100 mg, 0.260 mmol, 1 equiv) in 20 mL HPLC grade MeOH was bubbled with NH_3_ gas for 2 h with continuous stirring on an ice-water bath. A solution of hydroxylamine-*O*-sulfonic acid (98.1 mg, 0.868 mmol, 3.3 equiv) in 5 mL HPLC grade methanol was added dropwise via syringe, then the mixture was allowed to warm to room temperature and stirred for an additional 3 h. The crude reaction mixture was filtered and the filter cake was washed with MeOH. The combined filtrate was supplemented with 0.5 mL triethylamine and concentrated under reduced pressure. The white residue was dissolved in 9 mL MeOH and 1 mL triethylamine, then 10% iodine in methanol was added dropwise until the yellow color persisted (<1 mL). After excess iodine was removed by addition of solid sodium thiosulfate, the solution was concentrated *in vacuo*. The crude product was purified by flash column chromatography (CombiFlash, 10–50% hexanes/EtOAc over 15 min) to give photocholesterol as a white solid (17.0 mg, 0.0427 mmol, 17% yield).

^1^H NMR (400 MHz, CDCl_3_): 5.35 (dt, *J* = 5.0, 1.9 Hz, 1H, C6-H), 3.52 (q, *J* = 8.6, 5.8 Hz, 1H, C3-H), 2.34–2.18 (m, 2H, C4-H), 2.04–1.93 (m, 2H, C2-H, C12-H), 1.83 (m, 3H, C1-H, C7-H, C16-H), 1.62–1.03 (m, 17H, C2-H’, C7-H’, C11-H, C15-H, C8-H, C24-H, C12-H’, C16-H’, C17-H, C23-H, C22-H, C1-H’), 1.00 (s, 3H, C21-H), 0.99 (s, 2H, C19-H), 0.98–0.91 (m, 2H, C14-H, C9-H), 0.89 (d, *J* = 6.5 Hz, 3H, C26-H), 0.67 (s, 3H, C18-H).

^13^C NMR (100 MHz, CDCl_3_): 140.75, 121.68, 71.79, 56.73, 55.86, 50.09, 42.31, 42.30, 39.74, 37.24, 36.49, 35.63, 35.47, 34.72, 31.89, 31.66, 28.20, 25.96, 24.26, 21.07, 20.55, 19.96, 19.40, 18.60, 11.84.

MS–EI (*m/z*): calc’d for C_26_H_41_O+ [M+H-N_2_H_2_]+: 369.3157, found: 369.3150.

TLC (SiO_2_, 50% EtOAc–hexanes), R_*f*_: 0.42 (*p*-anisaldehyde, PMA)

### Construction of the fHog plasmid

All PCR reactions were performed using Phusion High Fidelity polymerase (NEB, M0530), and parent constructs were digested with DpnI (NEB, R0176). A region including the *Drosophila melanogaster* Hog domain (fHog, residues 244–471) was cloned into a pET-28a vector between a N- and C-terminal SUMO-His_6_ fusion tags. Briefly, the *D*. *melanogaster* Hog sequence was amplified from the pAct5C-Hh plasmid (Addgene #37382) by PCR using a forward primer containing a 5’ BamHI restriction site (5’-gggcccggatcctccgtcaagtcagattcg-3’) and a reverse primer containing a 3’ NotI restriction site (5’-accaccgcggccgcatcgtggcgccagctctgcgg-3’). The *D*. *melanogaster* Hog PCR product was digested with BamHI-HF (NEB, R3136) and NotI-HF (NEB, R3189) for 3 h at 37°C, purified by electrophoresis on 1% agarose, and isolated by gel extraction using a QIAquick gel extraction kit (Qiagen). A sequence for the C-terminal SUMO-His_6_ fusion tag was amplified by PCR from the pETDuet1-SUMO plasmid (gift from A. Hoelz) using a forward primer (5’-ataagaatgcggccgcactggaagttctgtttcaaggtccgatgtcggactcagaagtc-3’) containing a 5’ NotI restriction site followed by a PreScission protease cleavage site, and the reverse primer (5’-ccgctcgagaatctgttctctgtgagcctc-3’) containing a 3’ XhoI site. The C-terminal PreScission-SUMO insert PCR product was digested with XhoI (NEB, R0146) and NotI-HF for 3 h at 37°C and purified by electrophoresis as above. The backbone plasmid pET28a SUMO Nup53xl 1–320 (Addgene #85998) was digested with BamHI-HF and XhoI for 3 h at 37°C. The digested plasmid backbone, the *D*. *melanogaster* Hog insert, and the C-terminal PreScission-SUMO insert were then ligated using T4 ligase (NEB, M0318) at room temperature for 1.5 h. The resulting pET-28a plasmid containing fHog (His_6_-SUMO-fHog-PreScission-SUMO-His_6_), encoding 14 residues of the N-terminus and the entire C-terminus of the *D*. *melanogaster* Hedgehog protein flanked by N- and C-terminal SUMO-His_6_ tags, was verified by Sanger sequencing.

### Expression and purification of fHog protein

The pET-28a plasmid containing the fHog construct was transformed into *E*. *coli* BL21-CodonPlus (DE3)-RIPL cells (Agilent, 230280). Cells were grown in LB Broth (Lennox) (Sigma-Aldrich, L3022) containing 50 μg/mL kanamycin at 37°C and 225 rpm. Upon reaching an OD_600_ of 0.8, cells were induced with 1 mM isopropyl β-D-1-thiogalactopyranoside (IPTG, Chem-Impex, 00194), and grown for an additional 16 h at 18°C and 225 rpm. Cells were harvested by centrifugation, and pellets were flash frozen and stored at –80°C until purification.

*All protein isolation procedures were performed on ice or at 4°C*. The cell pellet from 3 L of *E*. *coli* growth was suspended in 50 mL of resuspension buffer (50 mM Tris-HCl pH 8.0, 500 mM NaCl, 5% glycerol) containing 50 μg/mL lysozyme (Alfa Aesar, J60701), 1× cOmplete EDTA-free Protease Inhibitor Cocktail (Roche, 11836170001), and 1 mM PMSF. Cells were lysed using a microfluidizer (Microfluidics, LM10), then lysate was supplemented with 1% Triton X-100 and incubated at 4°C for 45 minutes with gentle stirring. Cell debris was removed by centrifugation for 45 min at 27,300 × g at 4°C. Supernatant was carefully collected and filtered on ice through a 0.22 μm membrane filter before loading on two tandem 1-mL His-Trap high performance columns (GE Healthcare, GE17-5247) pre-equilibrated with wash buffer (50 mM Tris-HCl pH 8.0, 500 mM NaCl, 5% glycerol and 0.1% Fos-Choline (Anatrace, F308S)). The column was washed with 5 column volumes of wash buffer and protein was eluted using gradient elution (3–70%) with elution buffer (50 mM Tris-HCl pH 8.0, 500 mM NaCl, 500 mM Imidazole, 5% glycerol and 0.1% Fos-Choline). Peak elution fractions were collected and analyzed by SDS-PAGE, and the buffer was exchanged to low-salt buffer (25 mM Tris-HCl pH 8.0, 100 mM NaCl, 5% glycerol, 0.1% Fos-Choline) using an Econo-Pac 10DG desalting column (Bio-Rad, 7322010). The desalted fHog protein was further purified using an anion exchange column (HiTrap Q HP, GE Healthcare) by washing with 5 column volumes of low-salt buffer before eluting with 0–50% gradient of high-salt buffer (25 mM Tris-HCl pH 8.0, 2 M NaCl, 5% glycerol, and 0.1% Fos-Choline). The eluted sample was concentrated and exchanged into fHog buffer (25 mM Tris-HCl pH 8.0, 200 mM NaCl, 5% glycerol, and 0.1% Fos-choline) using a 10 kDa molecular weight cutoff centrifugal filter (Amicon, UFC801008) to a concentration of ~25 mg/mL, determined using a Pierce BCA Protein Assay Kit (Thermo Fisher Scientific, 23227). Protein samples were aliquoted, flash frozen in liquid nitrogen, and stored at –80°C.

### *In vitro* cholesterolysis

The purified fHog protein was thawed on ice, diluted to 6.25 μg/μL in activity buffer (20 mM HEPES pH 7.5, 50 mM NaCl, 0.1% Fos-choline, and 1 mM DTT), and aliquoted into a PCR tube strip. 2-Propanol alone or 50 μM photocholesterol in 2-propanol was added to a final concentration of 0.5 mM with continuous vortexing. The reactions was incubated with gentle rocking at room temperature for 4 h, then quenched by heating to 98°C for 5 min and adding 1× SDS-PAGE sample buffer. Samples were run on a Stain-Free 4–15% SDS-PAGE gel (Bio-Rad, 5678084) and visualized after UV activation on a Bio-Rad Chemidoc MP Imaging System. Each reaction was performed in triplicate.

### Photocholesterol crosslinking and trypsin digestion

Purified fHog protein at 50 μM in activity buffer (20 mM HEPES pH 7.5, 50 mM NaCl, 0.1% Fos-choline, and 1 mM DTT) containing either 0.5 mM photocholesterol (from a 50 mM solution in 2-propanol) or 2-propanol alone (to 1% final) were incubated on ice in 1.5 mL Eppendorf tubes for 4 h in the dark. Open tubes were then exposed to 368 nm light in a UV crosslinker (Fisher Scientific, 13-245-221) on ice for 5 min to a total of 600200 μJ. Samples were supplemented with 1× SDS-PAGE sample buffer and heated to 98°C for 5 min before loading on a 4–15% SDS-PAGE gel (Bio-Rad, 5678084). To isolate full-length fHog protein, the gel was stained with InstantBlue^®^ Coomassie protein stain (Expedeon, ISB1L) and bands at the apparent molecular weight of the SUMO-fHog-SUMO protein (~52 kDa) were excised and sliced into 0.5–1 mm^3^ cubes. The photocholesterol-fHog crosslinking experiment was performed in biological triplicate.

Gel cubes from each sample were washed 3× with 250 μL of 100 mM NH_4_HCO_3_ for 10 min, followed by 250 μL 1:1 (v/v) 50 mM NH_4_HCO_3_/acetonitrile (ACN) for 10 min. 500 μL ACN was added and the gel pieces were incubated at room temperature with occasional vortexing for 10 min. Gel pieces from each sample were incubated with 50 μL of 50 mM NH_4_HCO_3_ and 100 μL of 10 mM DTT at 50°C for 30 min on a mixer (Eppendorf ThermoMixer 5350). The DTT solution was removed with a pipette and the gel pieces were incubated with 50 μL of 50 mM NH_4_HCO_3_ and 100 μL of 55 mM iodoacetamide at room temperature for 30 min on a mixer in the dark. The gel pieces were washed with 250 μL 50 mM NH_4_HCO_3_ then with 250 μL ACN on a mixer for 5 min until slices were completely dehydrated, then dried using a Centrivap concentrator (Labconco, 7310022) for 2–3 min. Samples were placed on ice and covered with 60 μL of 6 ng/μL trypsin (Promega, V5113) in NH_4_HCO_3_ for 60 min. 20 μL of 50 mM NH_4_HCO_3_ was added and the samples were incubated overnight at 37°C on a mixer. The supernatant was removed and stored. The gel pieces were incubated sequentially with 200 μL of 1% formic acid/2% ACN, ACN/water, and 1% formic acid in ACN for 20 min each on a mixer and the supernatants were combined. The extracted peptides were dried for 3–4.5 h on a Centrivap concentrator. Peptides were resuspended in 10 μL 0.2% formic acid/2% ACN by vortexing and sonicating in a sonicator bath for 3 min. A C18 ZipTip (Thermo Scientific, 87784) was washed with 10 μL ACN followed by 10 μL 0.2% formic acid/2% ACN 3 times. Sample were desalted using the ZipTip and dispensed 20 times, then washed 4 times with 0.2% formic acid/2% ACN. The peptides were eluted into a 1.7 mL tube with 10 μL 50% ACN/0.2% formic acid five times followed by 10 μL 75% ACN/0.2% formic acid five times. The peptides were dried in the Centrivap concentrator and stored at –20°C. Peptides were resuspended in 0.2% formic acid/2% ACN for LC-MS analysis.

### Protein sequence analysis by LC-MS/MS

The desalted samples were resuspended in 2% ACN, 0.2% formic acid in water (10 μL), and 1 μL was subjected to LC-MS/MS analysis on an EASY-nLC 1200 (ThermoFisher Scientific, San Jose, CA) coupled to an Orbitrap Q Exactive HF mass spectrometer (Thermo Fisher Scientific, Bremen, Germany) equipped with a Nanospray Flex ion source. Samples were directly loaded onto a C18 Aurora column (25 cm x 50 μm ID, 1.6 μm, Ion Opticks, Parkville, Australia) maintained at 50°C and separated over 75 min at a flow rate of 350 nL/min with the following gradient: 2–6% Solvent B (3.5 min), 6–25% B (42 min), 25–40% B (14.5 min), 40–98% B (1 min), and 98% B (14 min). Solvent A consisted of 2% ACN, 97.8% H_2_O and 0.2% formic acid and solvent B consisted of 80% ACN, 19.8% H_2_O, and 0.2% formic acid. The Q Exactive HF was operated in data dependent mode with Tune (version 2.8) instrument control software. Spray voltage was set to 1.5 kV, S-lens RF level at 60, and heated capillary at 275°C. MS1 spectra were acquired at 60K resolution with a scan range from 400–1650 m/z, an AGC target of 3e6, and a maximum injection rate of 15 ms in Profile mode. A Top12 DDA analysis was then performed in which features were filtered for monoisotopic peaks with a charge state of 2–5, a minimum intensity of 1e5, and a minimum AGC target of 4.5e3, with dynamic exclusion set to exclude features after 1 time for 45 seconds and exclude isotopes turned on. HCD fragmentation was performed with normalized collision energy of 28 after quadrupole isolation of features using an isolation window of 1.2 m/z, an AGC target of 1e5, and a maximum injection time of 45 ms. MS2 scans were then acquired at 30K resolution in Centroid mode with the first mass fixed at 100 and a scan range of 200–2000 m/z.

### Proteomics data analysis

Data analysis was performed with Proteome Discoverer version 2.4. Spectra were filtered to HCD spectra with signal-to-noise > 1.5. Spectra were searched against a custom fasta file containing the recombinant protein sequence, SUMO-fHog-SUMO, and a database common contaminant proteins using SEQUEST HT. In SEQUEST HT, the search was conducted with mass tolerance of 10 ppm for the precursor and 0.02 Da for fragment ions from HCD, with semi-specific digestion, 2 missed cleavages, and a maximum of 3 equal modifications and 4 dynamic modifications per peptide. Static modifications were carbamidomethylation of cysteine (+57.021464 Da). Oxidation on methionine residues (+15.994915 Da), methionine loss on protein N-termini (−131.040485 Da), methionine loss + acetylation on protein N-termini (−89.02992 Da), protein N-terminal acetylation (+42.010565 Da) and photo-cholesterol modification on every amino acids (+370.3236 Da), were dynamic modifications.

Assignments were validated with Percolator in which FDR (false discovery rate) were set at 0.01 (strict) and 0.05 (relaxed). Peptide and PSM FDRs were set at 0.01 (strict) and 0.05 (relaxed), with peptide confidence at least low, lower confidence peptides included, and minimum peptide length set at 4. All peptides containing the photo-cholesterol modification were manually examined to validate the spectral interpretations. The precursors of spectra with high confidence assignments (FDR < 1%) to photo-cholesterol-modified peptides were manually examined for isotopic pattern and match quality.

### Expression and analysis of hSHH mutants in HEK293T cells

hSHH cloning, protein expression, and Western blot analysis were performed according to previously described methods [17]. Wild type hSHH (residues 1–462) and mutants were cloned in the pCMV6 vector (Origene RC222175) with a stop codon inserted before the C-terminal Myc-DDK tag (see S1 Table). Proteins were expressed in adherent HEK-293T cells (ATCC, CRL-3216) seeded at a density of 200,000 cells/well in 12-well plates in high-glucose DMEM containing 10% fetal bovine serum (FBS, Gibco, 26140079) 24 h before transfection. After reaching approximately 80% confluence, cells were transfected with 0.75 μg hSHH plasmid precomplexed with polyethylenimine (PEI) at a ratio of 1:3 DNA:PEI (w:w). Cells were cultured for 42–48 h after transfection, rinsed with 200 μL PBS, and collected in 125 μL ice-cold lysis buffer (50 mM Tris-HCl (pH 7.4), 250 mM NaCl, 1% IGEPAL CA-630, 1× cOmplete EDTA-free Protease Inhibitor Cocktail (Roche, 11836170001), and 1 mM PMSF). Cells were lysed using a sonicator tip (Qsonica, 4418), supplemented with SDS-PAGE sample buffer, and heated to 98°C for 5 min prior to analysis or storage at –20°C. Protein expression was analyzed by Western Blot using a primary Shh N-terminus Antibody (E-1) (Santa Cruz Biotechnology, sc-365112) and a primary Shh C-Terminus Antibody (R&D Systems, AF445). Secondary antibodies Alexa Fluor 647 conjugated Donkey anti-Goat IgG (Invitrogen, A-21447) and Alexa Fluor 488 conjugated Donkey anti-Mouse IgG (Invitrogen, A-21202) were used to visualize full-length hSHH protein and cleavage products. Band intensities were quantified using Bio-Rad Image Lab Software v6.0. Briefly, bands corresponding to full-length and/or processed hSHH proteins in each lane were identified by the software and visually inspected for accuracy, and relative pixel intensities were measured for each band.

### Molecular dynamics simulations

In our full-atomistic simulations, all molecules including protein, POPC, cholesterol, and ions were parameterized using the CHARMM36m force field [19]. Water was described using the TIP3P model [20]. Partial charges for the G197-C198 thioester in the reactant and G197 oxyanion in the tetrahedral adduct were generated using the ParamChem server [21, 22]. During the 400 ns simulations, the temperature was maintained at 303 K using a Nosé-Hoover [23, 24] thermostat with a damping constant of 1.0 ps for temperature coupling, and the pressure was controlled at 1 bar using the Parrinello-Rahman barostat algorithm [25] with a 5.0 ps damping constant for the pressure coupling. Semi-isotropic pressure coupling was used throughout the calculations. The Lennard-Jones cutoff radius was 12 Å, where the truncated non-bonded forces at the cutoff distance was smoothly shifted to 0 after 10 Å using a force-switch function. Periodic boundary conditions were applied in all three directions. The Particle Mesh Ewald algorithm [26] with a real cutoff radius of 12 Å and a grid spacing of 1.2 Å was used to calculate the long-range coulombic interactions. A compressibility of 4.5 ×10^−5^ bar^-1^ was used along the xy-plane and the z axis to relax the box volume. In all of the above simulations, water OH-bonds were constrained by the SETTLE algorithm [27]. The remaining H-bonds were constrained using the P-LINCS algorithm [28]. A simulation time step of 2 fs was used for integrating the equation of motions. All simulations were performed using GROMACS-2019.4 [20, 29] and the constrained dynamics simulations were done using PLUMED-2.5 [31].

#### Apo Hog, cholesterol-Hog complex, and “flipped” complex

Structures were optimized using 1000 steps of energy minimization according to the GROMACS steepest descent algorithm [29, 30] followed by an MD simulation in a canonical ensemble, where the system was heated from 0 K to 303 K for 105 ps. Next, an MD simulation in an isobaric-isothermal ensemble was performed with positional restraints on heavy atoms using a force constant of 9.6 kcal.mol^-1^ Å^-2^ for 51 ns. The z-coordinates of the POPC headgroups and cholesterol hydroxyl groups were restrained inside the membrane with a force constant of ~2.4 kcal.mol^-1^Å^-2^, while the POPC/cholesterol molecules were allowed to move freely along the xy-plane. Restraints on the protein, POPC, and cholesterol were progressively reduced to 0 kcal.mol^-1^Å^-2^. The system was equilibrated by performing an MD simulation at a temperature of 303 K and pressure of 1 bar for 400 ns. These calculations were carried out for the cholesterol-Hog, ΔP392-A423 cholesterol-Hog, and flipped cholesterol-Hog systems.

#### Hog-cholesterol adduct

Using a distance constraint of 4 Å and a force of 0.48 kcal.mol^-1^.Å^-2^ between the cholesterol C3-OH and the D243 carboxylate, a ~200 ns constrained dynamics simulation was performed to migrate cholesterol to the Hint active site. During this process, a stable salt bridge between D243 and R385 was maintained with a distance constraint of 4 Å at a force of 1.43 kcal.mol^-1^.Å^-2^. The resulting structure was equilibrated for an additional 400 ns with a distance constraint of 1.2 Å at a force of 1.4 kcal.mol^-1^.Å^-2^ between the sulfur atom of C198 and the C3-OH of cholesterol. In the equilibrated structure, a covalent bond was formed between the C3 oxygen atom of cholesterol and the carbonyl carbon of G197, and the protonation state of D243 was adjusted. The energy of the adduct was minimized over 1000 steps using the steepest descent algorithm. A ~105 ps MD simulation in a canonical ensemble was used to heat the system from 0 K to 303 K, followed by a ~1 ns MD simulation in an isobaric-isothermal ensemble with positional restraints on the heavy atoms using a force constant of 9.6 kcal.mol^-1^ Å^-2^. The z-coordinates of the POPC headgroups and cholesterol hydroxyl groups were restrained inside the membrane with a force constant of ~2.4 kcal.mol^-1^Å^-2^ and the POPC/cholesterol molecules were allowed to move freely along the xy-plane. Throughout the calculation, restraints on the protein, POPC, and cholesterol were reduced to 0 kcal.mol^-1^Å^-2^. The system was equilibrated in a ~400 ns MD simulation at a temperature of 303 K and pressure of 1 bar.

#### hSHH mutants

To analyze hSHH mutants were modeled by modifying optimized structures using the Dunbrack library [31] in UCSF Chimera [32, 33]. Each mutant structure was optimized for ~1 ns using positional restraints, followed by ~400 ns MD without restraints.

## Results

### MD simulations place a hSHH Hog protein on the membrane

Although the Hh cholesterolysis reaction was identified over 20 years ago [11], the structural details of Hog-promoted cholesterol ligation remain vague. In 2020, a first computational study by Banavali advanced a model of the full-length *D*. *melanogaster* (fly) protein using a cholesterol-binding bacterial cryptogein as a template for the SRR (S1 Fig) [34]. This study provided a detailed picture of the Hint fold active site during the cholesterolysis process, and suggested SRR residues that might influence the efficiency of the reaction. However, this model was generated in the absence of a membrane, and SRR residues that featured prominently in the fly model had little significance in our hSHH cholesterolysis experiments [17]. We therefore set out to construct an atomistic model of the human Sonic hedgehog Hog domain that incorporated our experimental results. In particular, we sought to address how the SRR associates with a cholesterol-containing membrane and coordinates with the Hint fold during cholesterolysis.

To define the relative orientation of the hSHH Hint fold (residues C198-A365) and the SRR (residues V366-S462), we performed mixed comparative/*ab initio* modeling of the hSHH Hog domain sequence using the Robetta server [35]. As a template for the well-characterized Hint fold, we employed a crystal structure of the *D*. *melanogaster* homologue after hydrolytic cleavage in the absence of a SRR [12]. Notably, studies on the hydrolysis of self-splicing inteins show that the Hint fold is largely unchanged during the splicing process, supporting the use of this template for our pre-cleavage model [36–39]. To evaluate *ab initio* structure predictions for the SRR, which has no homologues in the PDB, we relied on our biochemical and cellular analysis of hSHH SRR reactivity [17]. We eliminated structures that lacked α-helices that we had previously characterized by circular dichroism analysis (1^st^ SRR helix: W372-L390; 2^nd^ SRR helix: I432-L447). Likewise, we disfavored structures with significant contacts between the Hint fold and a non-conserved portion of the hSHH SRR loop (P392-A423), which we have shown to be dispensable for cholesterolysis. As we have observed that the SRR associates with cellular membranes, we selected structures in which one or both of the SRR helices had the potential to interact with a cholesterol-containing membrane. Finally, we favored models that positioned the SRR adjacent to the Hint active site (residues C198, T267, H271, and D243), since both the active site and the SRR are involved in cholesterolysis.

**Fig 2:**
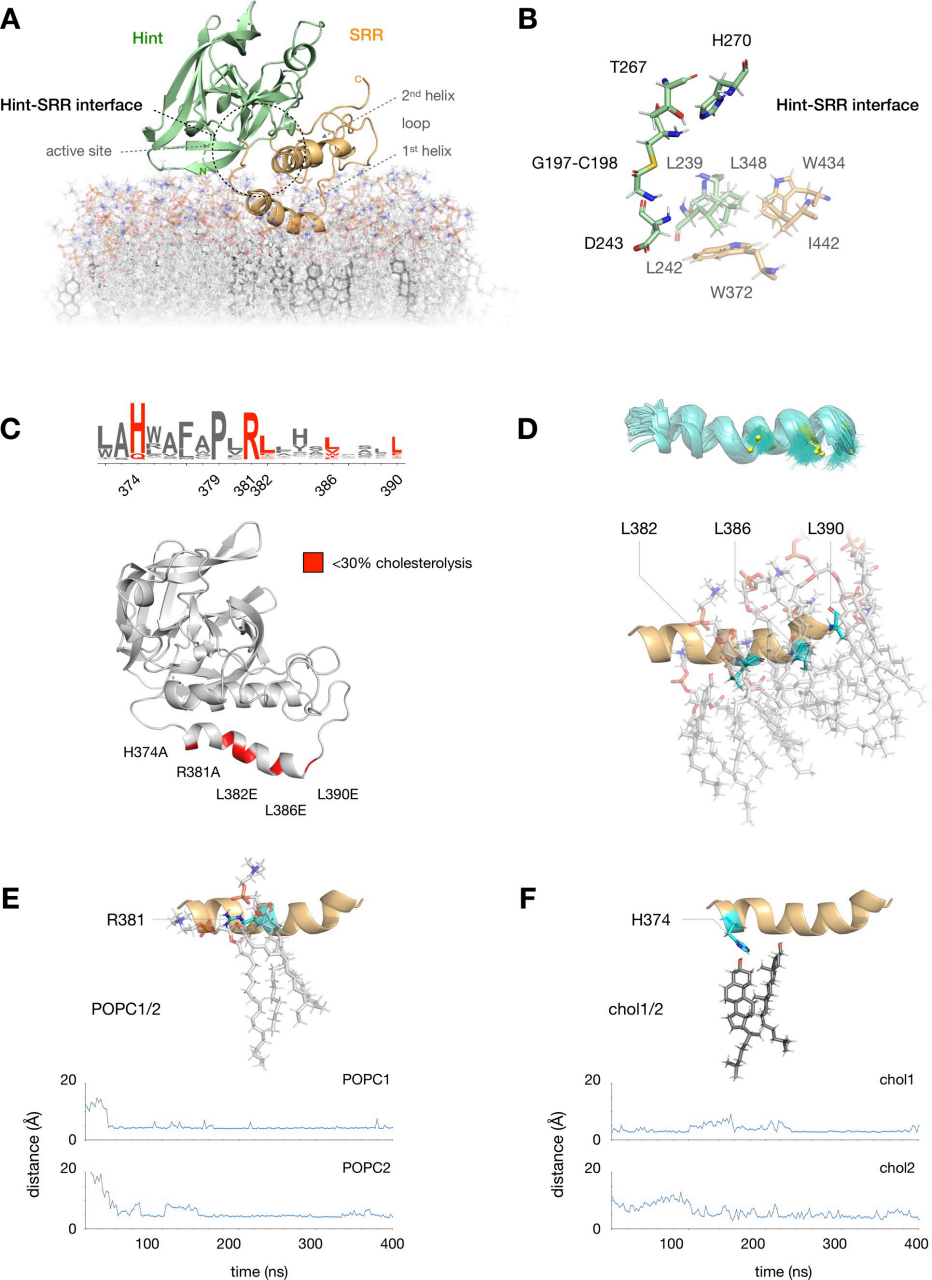
MD analysis highlights hydrophobic SRR interactions. (A) Equilibrated MD model of the apo hSHH Hog domain in a phosphatidylcholine (POPC) bilayer containing ~20% cholesterol; the Hint-SRR interface is indicated by a dashed black circle. (B) Expansion of residues near the Hint fold active site (D243, T267, H270, and the G197-C198 thioester). Hydrophobic residues in the Hint fold (L239, L242, and L348) and the SRR (W372, W434, and I442) remain within 5 Å of each other during a 400 ns MD equilibration. (C) Summary of conserved residues in the 1^st^ SRR helix (W372-L390) where mutation impairs hSHH cholesterolysis in cells (ref 17). (D) Three leucine residues in the 1^st^ SRR helix (L382, L386, and L390) remain localized next to POPC tails during equilibration. Top: superimposed trajectories (teal) of L382, L386, and L390 side chains (yellow) over 400 ns. Bottom: 400 ns snapshot of the 1^st^ SRR helix, showing L382, L386, and L390 (teal) interacting with POPC tails in the membrane. (E) Above: 400 ns snapshot of the interaction between R381 (teal) and two POPC molecules. Below: Plot showing equilibration of the distance between the R381 side chain and POPC headgroups to 3.9 ± 0.4 Å (POPC1) and 4.7 ± 1.8 Å (POC2) over 400 ns. (F) Above: 400 ns snapshot of the interaction between H374 (teal) and two cholesterol molecules. Below: Plot showing equilibration of the distances between the H374 side chain and two cholesterol C3-OH groups to 3.9 ± 0.4 Å (chol1) and 4.7 ± 1.8 Å (chol2) over 400 ns.

To address potential membrane interactions in our analysis, we placed our top Robetta model in a POPC membrane using the CHARMM-HMMM server [40]. With the resulting approximation, we created a full lipid bilayer containing 100 POPC molecules in each leaflet with ~20% cholesterol content (24 cholesterol molecules in each leaflet), approximating the composition of cholesterol-rich microdomains in the Golgi and endoplasmic reticulum [41–44]. Several previous studies have demonstrated that Hedge domain residues N-terminal to C198 are not required for cholesterolysis [45–48]; therefore, we modeled the N-terminal portion of hSHH (C24-G197) as a G197-C198 thioester preceded by a S195-G196 dipeptide for computational simplicity. To prepare the system for MD simulations, we solvated the protein, neutralized charges with NaCl (0.15 M), and adjusted the system to physiological pH (7.4). Using the steepest descent algorithm [29, 30] with positional restraints on heavy atoms, we minimized the energy of the system while allowing the POPC/cholesterol molecules to move freely along the xy-plane (see Materials and Methods) [31, 32]. We then gradually removed restraints on the protein, POPC, and cholesterol, and performed a ~400 ns equilibration of the “apo” Hog structure (Fig 2A).

Compared to our initial model, MD equilibration resulted in separation of the two SRR helices in the “apo” structure from a closest Cα-to-Cα distance of ~6.2 Å to ~8.1 Å (S2 Fig). In the process, the 2^nd^ SRR helix rotated approximately 45° toward to the 1^st^ helix to assume an antiparallel arrangement in the helix-loop-helix motif. These movements created an extended interface between residues adjacent to the active site in the Hint fold (L239, L242, L348) and residues in the 1^st^ (W372) and 2^nd^ (I442) SRR helices (Fig 2B). At the same time, the Hint fold migrated approximately 10 Å from the membrane surface, consistent with the reported solubility of the Hint fragment alone [12].

Notably, the 1^st^ SRR helix remained firmly anchored to the surface of the phospholipid bilayer throughout the simulations. Our previous studies showed that the 1^st^ SRR helix in hSHH contains a conserved HPR motif (H374, P379, R381) succeeded by three leucine residues (L382, L386, L390) that are critical for hSHH cholesterolysis in cells (Fig 2C) [17]. The requirement for these leucine residues in cellular but not biochemical activity suggests that they might play a role in membrane association. Consistent with this proposal, CHARMM-HMMM analysis spontaneously embedded the L382, L386, and L390 isobutyl side chains in the membrane, where they formed persistent hydrophobic interactions with POPC lipid tails over the course of our MD simulations (Fig 2D). Reinforcing this hydrophobic interaction was a stable salt bridge between the R381 guanidinium side chain and adjacent POPC headgroups (Fig 2E).

Our analysis also identified two molecules of cholesterol in the membrane that remained below the 1^st^ SRR helix throughout MD equilibration (Fig 2F). This localization was enforced by persistent H-bond and polar interactions between the cholesterol C3-OH groups and the imidazolium side chain of H374. To determine if this interaction might help to sequester membrane cholesterol, we computationally generated an H374A mutant and equilibrated the system for 400 ns (S3 Fig). Over the course of the simulation, the two localized cholesterol molecules strayed from their positions beneath the 1^st^ SRR helix, showing little positional bias over molecules at distant locations. Combined with our experimental analysis, these results suggest a mechanism by which the 1^st^ SRR helix can bias localization of cholesterol in the upper leaflet, recruiting it to the Hog protein.

### Photocholesterol captures a non-covalent binding site

**Fig 3:**
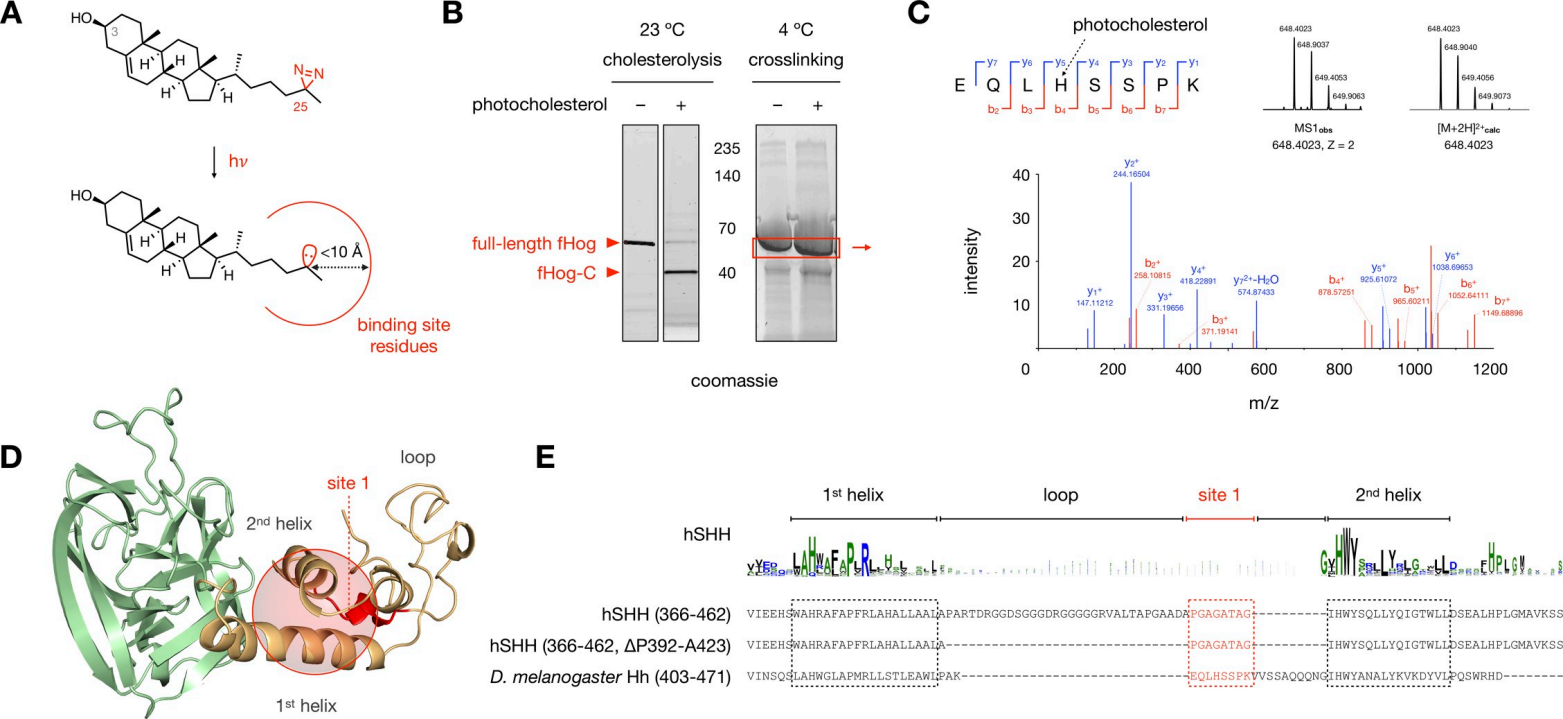
Photocholesterol labels an essential region of the SRR loop. (A) Activation of the C25 diazirine in photocholesterol with 368 nm UV light yields a reactive carbene with a crosslinking radius of <10 Å (ref 51). (B) Left: Stain-Free gel showing cholesterolysis of an aliquot of fHog protein. (1 mM DTT, ± 50 μM photocholesterol, 23°C, 4 h). Right: Representative Coomassie-stained gel showing fHog protein incubated with photocholesterol or vehicle (1 mM DTT, ± 50 μM photocholesterol, 0°C, 4 h) and exposed to 368 nM light (5 min, 0°C). Protein in the red box was excised, digested with trypsin, and analyzed by mass spectrometry. (C) Mass spectrum of a crosslinked peptide at site 1; the photocholesterol-modified histidine residue is indicated by a dashed black arrow. For full assignments, see S1 File. (D) A sphere encompassing residues within 10 Å of the loop identified by site 1 (red) in the hSHH apo Hog model. (E) Alignment of the helix-loop-helix motifs in fHog and hSHH shows putative homology between the fHog crosslinking site 1 and the 8 residues of the hSHH SRR loop that are required for cellular cholesterolysis. fHog cholesterolysis with photocholesterol and crosslinking/mass spectrometry were performed in biological triplicate.

Because cholesterol cannot access the internal thioester directly from the membrane, we hypothesized that conserved hydrophobic residues in the Hog domain [49] might act to shield cholesterol on its route to the G197-C198 thioester. To address this possibility, we synthesized a cholesterol photoaffinity probe bearing a diazirine at the terminal carbon of the iso-octyl tail (C25) (Fig 3A) [50]. This modification enables UV light-induced formation of a C25 carbene, which can crosslink to residue side chains with a radius of a few angstroms [51]. The formation of a covalent bond between C25 and residues in the immediate vicinity enables isolation and mass spectrometric analysis of corresponding non-covalent interactions with cholesterol. Importantly, replacement of the C25 dimethyl unit with a diazirine has a minimal effect on the steric properties, polarity, and lipid interactions of the natural molecule.

To generate sufficient quantities of functional Hog protein for crosslinking/mass spectrometry analysis, we designed and expressed an active Hog construct in *E*. *coli*. For these studies, we used the *D*. *melanogaster* Hog protein (244–471), which is amenable to *in vitro* expression [12, 53]. To facilitate purification and limit off-target crosslinking by photocholesterol, we introduced relatively small (11 kDa) N- and C-terminal SUMO proteins, yielding a SUMO-Hh(244–471)-SUMO (“fHog”) fusion protein. Analysis of detergent-solubilized fHog protein revealed that cholesterolysis reached 86 ± 4% completion after a 4 h incubation with 50 μM photocholesterol at room temperature (Fig 3B). To identify non-covalent cholesterol-Hog interactions by photoaffinity crosslinking, we incubated the fHog protein with photocholesterol or vehicle at 4°C in the dark, which prevented covalent adduction. To instantaneously capture non-covalent interactions, we maintained the protein on ice and initiated carbene formation with 368 nm light for 5 min. Gel electrophoresis and isolation of the crosslinked full-length fHog protein by gel excision, followed by trypsin digestion and mass spectrometry analysis, provided 100% coverage of the fHog protein in each sample. In the photocholesterol-treated samples, tandem MS/MS analysis identified peptides at one site in the SRR (site 1) and two sites in the Hint fold (sites 2 and 3) with the exact mass of the photocholesterol modification (Fig 3C and S4 Fig).

A peptide identified in three biological replicates was located at site 1, in the SRR loop at the back of the cholesterol binding pocket (Fig 3C and 3D). Consistent with the residue-agnostic nature of diazirine crosslinking, we observed product ion spectra that suggested modification at E, L, or H (e.g. Fig 3C) within this peptide (S1 File). While the Hh loop region varies widely in length and composition among species and isoforms, a flexible linker between two helices is universally maintained. Previously, we identified a minimum tether sequence between the 1^st^ and 2^nd^ helices in the hSHH SRR comprised of only eight (P392/424 to G431) of the 40 residues in the wild type protein (Fig 3E) [17]. Compellingly, when aligned by the SRR helix-loop-helix motif, crosslinking site 1 in fHog maps to the essential 8-residue piece of the hSHH SRR loop. The identification of multiple photocholesterol-modified peptides in the SRR loop suggests a possible orientation for cholesterol at the Hint-SRR interface.

### Mutations at the Hint-SRR interface disrupt cholesterolysis

**Fig 4:**
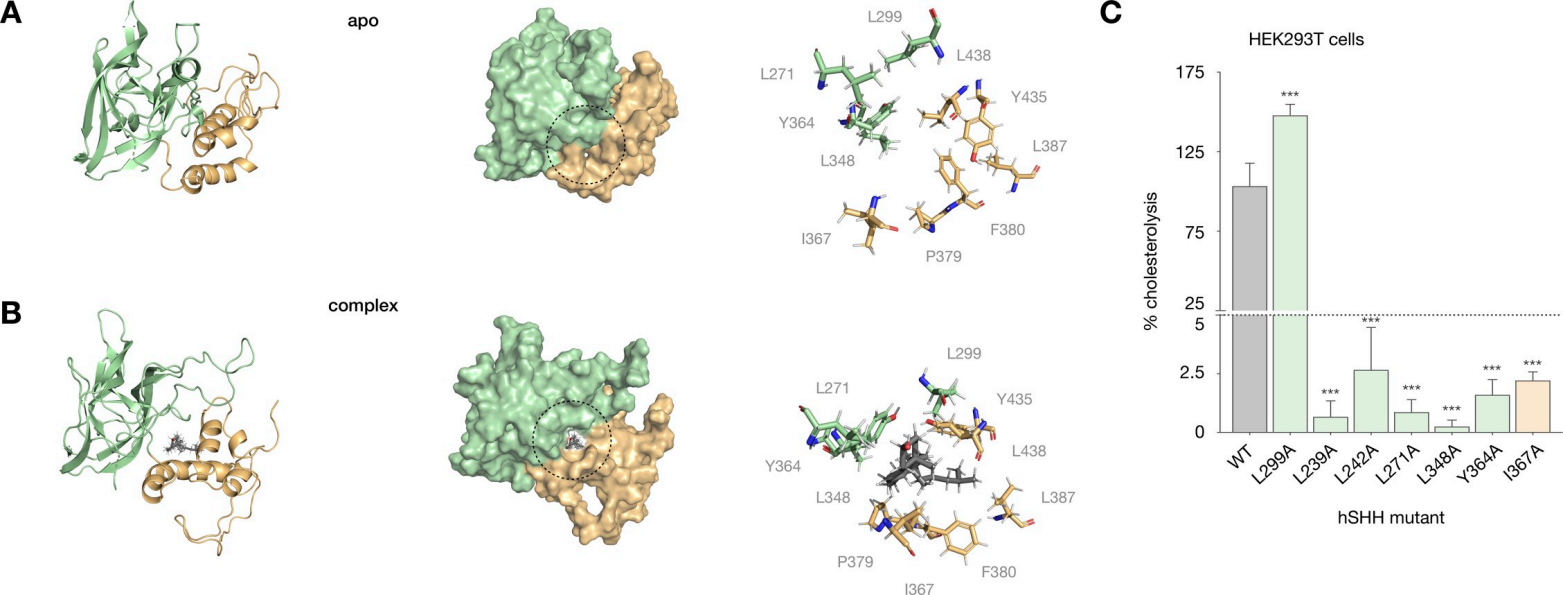
Binding site residues in a cholesterol-Hog complex affect cellular cholesterolysis. (A) Cartoon and surface representations of the apo hSHH Hog model; the Hint-SRR-cholesterol interface is indicated by a dashed black circle. (B) Cartoon and surface representations of the cholesterol-Hog complex, showing cholesterol non-covalently bound to the Hint-SRR interface. For A and B, the expansions shows residues that remain within 5 Å of cholesterol in the cholesterol-Hog complex during a 400 ns MD equilibration. (C) Relative cholesterolysis efficiency of full-length hSHH and mutant proteins overexpressed in HEK293T cells, evaluated by quantitative Western blot. Values are the average of 3–9 biological replicates ± s.d, one-way ANOVA, F(6,37) = 240.9, P value P < 0.001, Dunnett’s multiple comparisons test, a = 0.05, ***p < 0.001.

Guided by our photocholesterol crosslinking analysis, we docked cholesterol with its C25 tail within crosslinking distance of the identified 8-residue sequence. In our model, the sequence identified by crosslinking lies near the HWY hinge at the closest point between the 1^st^ and 2^nd^ SRR helices. This constraint oriented the C3-OH group of cholesterol toward the Hint active site with the hydrophobic core aligned with the Hint-SRR interface (Fig 3D). We evaluated the resulting binding pose using MD simulations to survey low-energy cholesterol-complexed structures. After an aggregate ~700 ns MD simulations to relax and optimize the system, cholesterol established a stable hydrophobic interaction network at the Hint-SRR interface (Fig 4A and 4B; see Materials and Methods). During equilibration, the C-terminus of the 1^st^ SRR helix reoriented toward the active site, while the N-terminus moved away from the Hint fold to accommodate cholesterol. Within the Hint fold, three leucine residues adjacent to the active site (L239, L242, and L348) and nearby hydrophobic residues (L271, L299, and Y364) made multiple contacts to the cholesterol tetracycle. The cholesterol tail settled next to the H433-W434-Y435 (HWY) “hinge” next to the identified 8-residue segment of the SRR loop.

To test whether a deletion mutant containing only the identified 8-residue loop sequence (P392/424 to G431) would also accommodate cholesterol in this pose, we removed the 32 non-essential loop residues from the apo Hog model, formed a bond between P392 and G431, and equilibrated the system in MD simulations for 400 ns. In line with our experimental data, the deletion mutant preserved association of the 1^st^ SRR helix with the membrane, interactions between the Hint fold and the SRR, and the relative orientation of the two SRR helices. Critically, docking of cholesterol in the mutant model placed C25 tail within crosslinking distance of the 8-residue sequence identified in our crosslinking analysis (S4 Fig).

In line with their potential roles in our model, hydrophobic residues at the Hint-SRR interface (L239, L242, and L271) and in the cholesterol binding site (L348, Y364, and L299) are conserved in Hh proteins. To assess the roles of these residues in cholesterolysis, we mutated each to alanine expressed their full-length hSHH proteins in human embryonic kidney (HEK293T) cells. Our analysis revealed that single alanine mutations at 6/7 residues (L239A, L242A, L271A, L348A, Y364A, and I367A) reduced cholesterolysis to <5% of the wild-type protein (Fig 4C). By contrast, a L299A mutant increased cholesterolysis by ~50%. In our model, the L299 backbone carbonyl forms a water-mediated H-bond to the C3-OH of cholesterol on a path to the G197-C198 thioester (S5A Fig). While a more detailed analysis of the trajectory is required, a smaller alanine residue at this position may provide greater access to the H-bond on the path to cholesterolysis.

The Hint-SRR interface in our model contains several aromatic residues (Y364, F380, Y435), which can engage in hydrophobic and/or π-stacking interactions to stabilize a cholesterol ligand [52]. In this and our previous study, we demonstrated that mutation of the conserved residues Y364 and F380 eliminated cholesterolysis of full-length hSHH protein in HEK293T cells. To assess the contribution of these residues to the stability of the cholesterol-complexed model, we replaced each with alanine and equilibrated the mutant models in MD simulations for 400 ns. Intriguingly, loss of a hydrophobic interaction between the cholesterol tail and the F380 side chain resulted in dissociation of cholesterol from the F380A Hog complex (S5B Fig). Likewise, equilibration of a Y364A mutant showed that loss of a potential π–π interaction with the cholesterol tetracycle eliminated cholesterol’s trajectory to the active site (S5C Fig). Our combined experiments demonstrate that: (1) mutation of cholesterol-binding residues in our model impairs cellular cholesterolysis, and (2) cholesterol dissociates from models of cholesterolysis-deficient mutants. Together, these observations provide complementary support for a cholesterol-Hog complex with a cholesterol binding site at the Hint-SRR interface.

### MD simulations show a dynamic path to adduction

**Fig 5:**
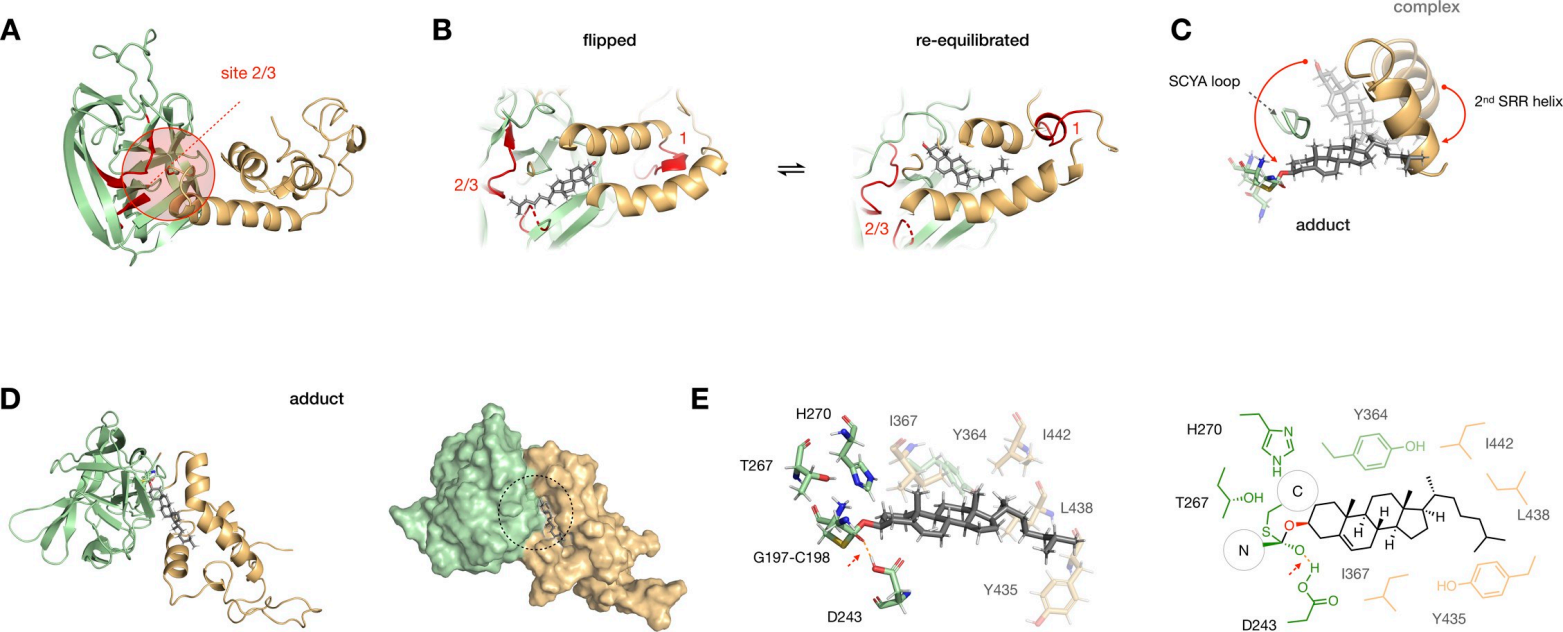
Photocrosslinking and MD analysis suggest dynamic cholesterol binding at the Hint-SRR interface. (A) A sphere encompassing residues within 10 Å of identified crosslinking sites 2 and 3 (red) in the hSHH apo Hog model. (B) Model of a “flipped” cholesterol-Hog complex, in which cholesterol is rotated 180° relative to its original pose. MD equilibration of the “flipped” cholesterol for 400 ns returns cholesterol to its original orientation (see S1 Movie). (C) Overlay of cholesterol, the 2^nd^ SRR helix, G197-C198 thioester, and SCYA loop in the cholesterol-Hog complex (semi-transparent) and the Hog-cholesterol adduct (full color). The red arrow indicates overall movement of cholesterol past the flexible SCYA loop during this transition (see S2 Movie). (D) Cartoon and surface representations of the Hog-cholesterol adduct; the thioacetal adduct is indicated by a black dashed circle. (E) Expansion of the thioacetal adduct, showing active site residues (full color) and Hint-SRR residues near cholesterol (semi-transparent). The H-bond between the G197 oxyanion and the protonated side chain of D243 is indicated by a dashed red line.

In addition to the peptides in the SRR loop, our crosslinking analysis identified photocholesterol-modified peptides at two sites in the Hint fold. The additional modified peptides encompassed active site residues C198 (C258 in *D*. *melanogaster*, site 2), and T267/H270 (T326/H329 in *D*. *melanogaster*, site 3) (Fig 5A and S1 File). To account for these crosslinked peptides, we envisioned that photocholesterol might also bind to the Hint-SRR interface in the opposite orientation. To evaluate a cholesterol-flipped pose computationally, we rotated cholesterol approximately 180° in its binding pocket and equilibrated the system in MD simulations for 400 ns (Fig 5B). Surprisingly, over the course of our analysis, cholesterol circumnavigated the binding pocket and re-established its original orientation. A movie of the equilibration process shows that cholesterol maintains continuous hydrophobic interactions with residues at the Hint-SRR interface (S1 Movie).

With this flexibility in mind, we examined possible paths for cholesterol to travel from the its position in the non-covalent complex to a position at the reaction site. Importantly, formation of a covalent bond to the Hh protein requires the C3-OH of cholesterol to approach the C197-C198 thioester at an appropriate angle for orbital overlap. To guide cholesterol relocation, we used a modest force constant (0.48 kcal.mol^-1^.Å^-2^) to place a distance constraint of 4 Å between C3-OH and D243 in the Hint fold active site (see Materials and Methods) [12, 53, 54]. A movie of cholesterol’s migration to the active site during constrained dynamics analysis shows that conformational changes in the loop and HWY hinge at the back of the cholesterol pocket orient cholesterol for attack (S2 Movie). As the 2^nd^ SRR helix swings ~45° closer to the Hint fold, the C-terminal segment of the 1^st^ helix rotates ~30° away, providing a broader opening near the G197-C198 thioester. These changes enable cholesterol to circumvent a conserved SCYA sequence (S362-E369) in a loop that connects the Hint fold and SRR. Concomitantly, the tail of cholesterol moves toward hydrophobic residues adjacent to the active site (Fig 5C). Significantly, we found that the distance between the sulfur atom of C198 and the C3-OH of cholesterol spontaneously equilibrated to ~4 Å, aligning cholesterol for covalent reaction.

To investigate the tetrahedral Hog-cholesterol adduct, we rehybridized the G197 carbonyl carbon from sp^2^ to sp^3^ and formed a carbon-oxygen bond to the C3-hydroxyl group of cholesterol. Performing a ~400 ns MD simulation with a 1.2 Å distance constraint between the bonded atoms provided a stable structure with cholesterol near the front of the Hint-SRR interface (Fig 5D). In this arrangement, cholesterol makes contacts to W372, F377, and F380 in the 1^st^ SRR helix, Y435, L438 L439, I442, and L446 in the 2^nd^ SRR helix, and L242, I367, and Y364 in the Hint fold (Fig 5E). Intriguingly, while we placed no constraints on the distance between the G197 oxyanion and the acidic proton of D243, these two atoms engaged in a 1.7 Å hydrogen bond over the course of equilibration (S6 Fig). This finding agrees with recent biochemical studies showing that a neutral D243 side chain stabilizes the oxyanion in during adduction, providing key insight into the mechanism of catalysis [53, 54].

### Disease-associated hSHH mutations displace cholesterol

In the developing embryo, the Hh morphogens are produced in specialized tissues and secreted to form a gradient that shapes the body plan [1, 2, 55]. Aberrant function of the hSHH protein is a leading cause of holoprosencephaly (HPE), a condition associated with improper formation of the embryonic forebrain [3–5, 56–59]. While high-resolution structures for the *D*. *melanogaster* Hint fold provide insight into disease-causing Hint mutations, the lack of an atomic hSHH SRR structure prevents such analysis for disease-causing mutations in the SRR. Our model of hSHH SRR provides an opportunity to identify congenital mutations that might interfere with cholesterolysis in disease.

**Fig 6:**
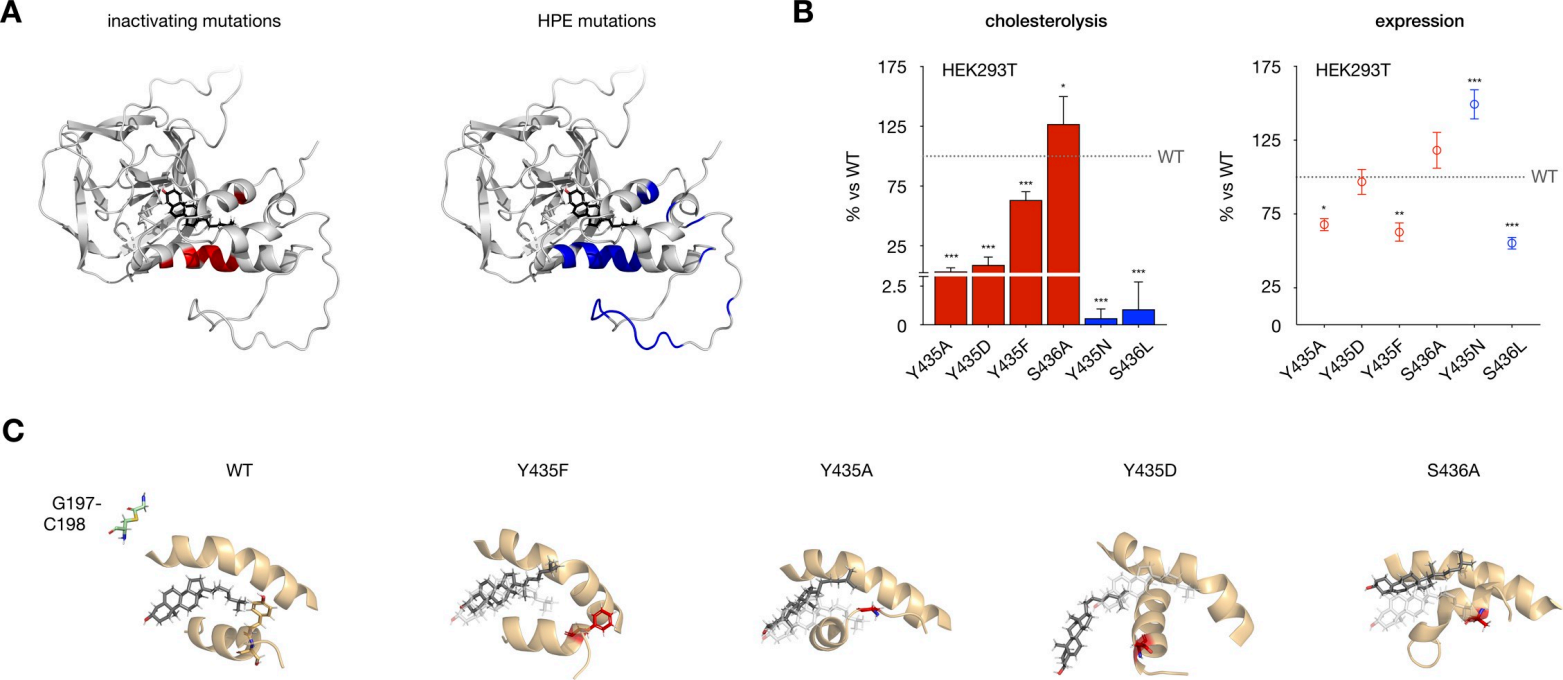
Disease-associated SRR mutations perturb hSHH cholesterolysis. (A) Model of the cholesterol-Hog complex, showing residues essential for cholesterolysis of full-length hSHH protein in HEK293T cells (red; see ref 17) and residues identified in holoprosencephaly (HPE) patients (blue). (B) Relative cholesterolysis efficiency and total expression of full-length hSHH and mutant proteins overexpressed in HEK293T cells, evaluated by quantitative Western blot. Values are the average of 3–9 biological replicates ± s.d. For cholesterolysis, one-way ANOVA, F(6,37) = 121.3, P value P < 0.001, Dunnett’s multiple comparisons test, a = 0.05, ***p < 0.001, *p = 0.02; for expression, one-way ANOVA, F(6,37) = 27.63, P value P < 0.001, Dunnett’s multiple comparisons test, a = 0.05, ***p < 0.001, **p = 0.003, *p = 0.02. (C) 400 ns MD-equilibrated of mutant complexes Y435F, Y435D, and Y435F show progressive displacement of cholesterol from its binding site, while an S436A mutation directs cholesterol toward the G197-C198 thioester.

A first analysis shows notable correlation between hSHH SRR mutations identified in HPE and residues that we have found to be essential for cholesterolysis (Fig 6A). To evaluate our hSHH Hog model in the context of clinical mutations, we examined two HPE-associated residues: Y435 and S436. Mutation at S436 (S436L) is associated with cyclopia, microcephaly, cleft palate, and chronic seizures [56], while symptoms of the Y435 mutation (Y435N) have not been reported [4]. In our cholesterol-complexed Hog model, Y435 and S436 appear at the conserved HWY motif in the hinge near the back of the cholesterol binding pocket.

Our previous studies have established that Y435 plays a critical role in the cholesterolysis reaction. However, while Y435A and Y435D mutations inhibit cellular cholesterolysis by >90%, a Y435F mutant retains >60% activity of the wild type protein (Fig 6B). In our cholesterol-complexed Hog model, Y435 lies close to a leucine-rich patch in the 1^st^ SRR helix (L386, L387, and L390), suggesting that it might engage these residues in hydrophobic and/or π interactions. To evaluate whether π interactions involving the Y435 aromatic ring might influence the cholesterol-Hog complex, we generated Y435A, Y435D, and Y435F cholesterol-complexed mutants and equilibrated them for 400 ns in MD simulations (Fig 6C). While mutation at Y435 to alanine and aspartate progressively displaced cholesterol from its binding site, Y435F showed only minor perturbations in structure versus the wild type complex. Preservation of the cholesterol complex by the aromatic residue in Y435F is consistent with a hypothesis that π interactions may contribute to the role of this residue in cholesterolysis.

Interestingly, while the S436 side chain makes no contacts to cholesterol or nearby residues in our model, a S436A mutant shows a 27% increase in cellular cholesterolysis compared to wild type protein (Fig 6B). When we equilibrated an S436A mutant for 400 ns in MD simulations, we found that cholesterol spontaneously moved below the S362-E369 loop, advancing it toward the Hint fold active site (Fig 6C). Identification of the same trajectory for cholesterol in our constrained dynamics analysis of thioester attack (Fig 5C and S2 Movie) suggests that this movement may reflect a thermodynamically feasible reaction path.

To examine the disease-associated mutations Y435N and S436L, we assessed their cholesterolysis activity in cells and the stability of their models during MD equilibration (Fig 6B). While both mutants reached less than 2.5% cholesterolysis of the wild type protein, the mutant models largely preserved the integrity of the cholesterol-Hog association. Interestingly, while the S436A mutant showed only 56% expression versus the wild type protein, a Y435N mutation increases total protein production to 149%. In these cases, protein folding, localization, or alternative pathways may play a dominant role in cellular cholesterolysis and/or disease. Our results suggest that combined experimental and computational approaches can inform the analysis of clinical SRR mutations.

## Discussion

Using molecular dynamics simulations, photocholesterol crosslinking/mass spectrometry analysis, and mutagenesis assays in cells, our studies provide evidence for a cholesterol binding site at a previously unrecognized Hint fold-SRR interface. Spontaneous association of hSHH Hog with a cholesterol-rich POPC membrane in our models suggests a path by which the SRR can localize cholesterol, form a non-covalent cholesterol complex with the Hint fold, and reorganize the binding site for reaction. Identification of clinical mutations at the Hint-SRR interface highlights the potential of such models to inform Hh-associated disease.

**Fig 7:**
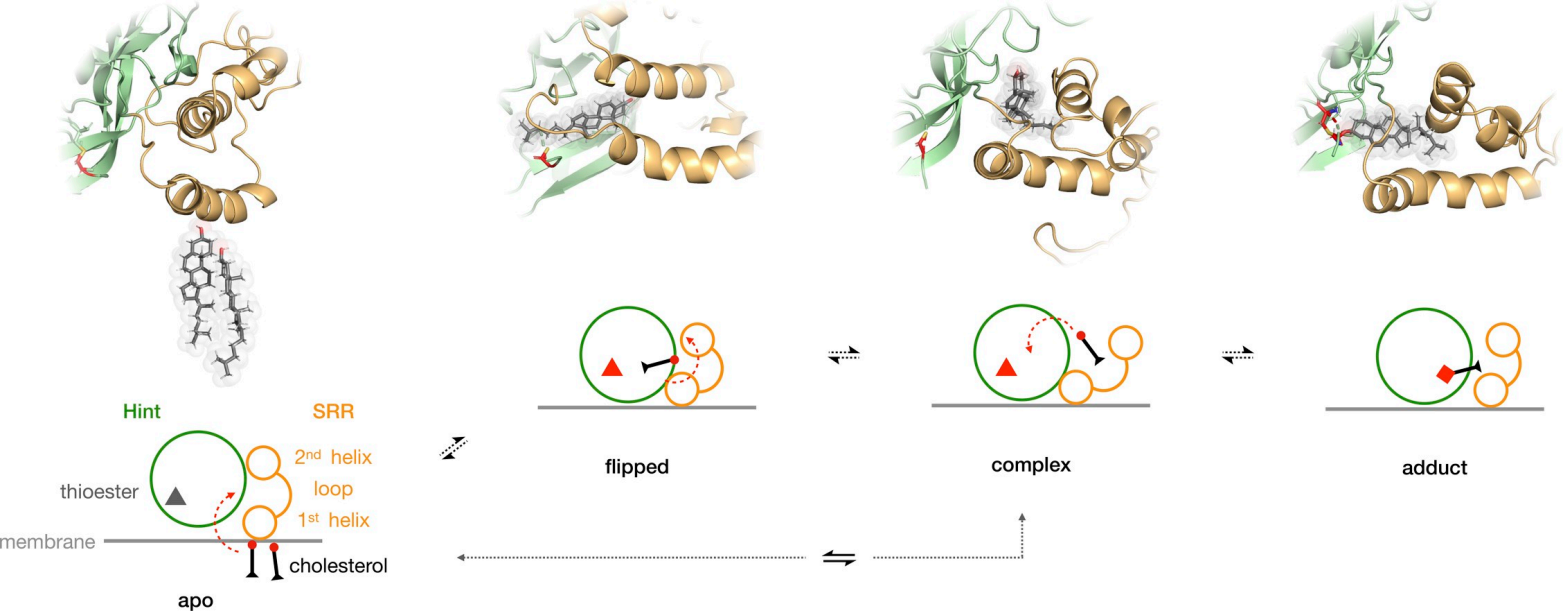
Cholesterol-Hog intermediates provide a path to covalent adduction. Modeled and schematic representations of Hh-cholesterol adduction. The 1^st^ helix of the SRR associates with the membrane and localizes cholesterol (apo). Reorientation of the SRR helices provides a hydrophobic pocket for cholesterol to equilibrate near the SRR loop (complex). *En route* to covalent bond formation, the 2^nd^ SRR helix migrates toward the Hint fold, and cholesterol moves to a position to attack the G197-C198 thioester. Addition of the C3-OH of cholesterol to the carbonyl of the G197-C198 thioester creates a covalent bond between cholesterol and hSHH (adduct). Molecular dynamics calculations suggest that cholesterol can “flip” head-to-tail through potential equilibration with apo and/or complexed structures.

Our models of the hSHH Hog domain in a membrane environment provides a sequence of viable intermediates *en route* to formation of the first covalent hSHH-cholesterol adduct (Fig 7). In the proposed scenario, associations between the membrane and residues in the 1^st^ SRR helix facilitate cholesterol recruitment. A hydrophobic Hint-SRR interface creates flexible cholesterol binding pocket, permitting equilibration between productive and flipped orientations. Conformational changes in the SRR enable cholesterol to come within bonding distance of the C197-C198 thioester, whereupon catalytic Hint residues facilitate formation of a tetrahedral intermediate. Energetically feasible structures at each node of the trajectory satisfy our crosslinking and mutagenesis data, and are consistent with established biochemical events that occur during cholesterolysis.

These atomistic techniques provide a unique window into dynamic protein assemblies. Further mapping of the cholesterol binding site using photocholesterol probes with alternative diazirine placement will create a more comprehensive picture of non-covalent interactions. Systematic *in vitro* assays using liposomes with defined compositions, sizes, and cholesterol content are required to better understand the influence of the membrane on cholesterol association and protein structure. We anticipate that approaches in diffraction-based analysis will provide new structural data regarding stable Hog assemblies. In a physiological context, work to understand the cues that inhibit or promote conformational changes will provide new insight into pathological Hh mutations and functions. Ultimately, a molecular analysis of protein structure and function can provide unique insight into the role of atomic forces in human health.

## Supporting information

S1 Fig Comparative/*ab initio* modeling reveals a Hint-SRR interface in hSHH. (A) Highest-scoring comparative/*ab initio* model from the Robetta server, using the crystal structure of *D*. *melanogaster* (PDB 1AT0) as a template for the hSHH Hint fold (residues 198–365) and *ab initio* modeling of the hSHH SRR (residues 366–462). (B) A model of the Hog domain from *D*. *melanogaster* Hh (C158-D471), from ref 32. In both models, the 1^st^ SRR helix in a helix-loop-helix motif is colored red; the 2^nd^ SRR helix is colored blue, the SRR loop residues are orange, and the Hint fold is green. Atoms in the active site cysteine residue (C198 in hSHH; C258 in *D*. *melanogaster* Hh) are represented as grey spheres.
(TIFF)

S2 Fig Molecular dynamics simulations refine the hSHH apo Hog structure in the membrane. Left: Top-scoring hSHH Hog model from Robetta docked at the membrane using the CHARMM-HMMM server. Right: Model after MD simulations (for details, see Materials and Methods). Red arrows highlight changes during MD simulations. The Hint fold migrates approximately 10 Å from the membrane (1), the 2^nd^ SRR helix rotates approximately 60° to assume an antiparallel arrangement with the 1^st^ SRR helix (2), and the two helices of the SRR separate from a closest Cα-to-Cα distance of 6.2 Å to a distance of 8.1 Å (3).
(TIFF)

S3 Fig A H374A mutant loses stable contacts to cholesterol. (A) Top: Superposition of 1^st^ SRR helix structures from 400 ns MD equilibration of the wild type apo Hog model. Bottom: Plot of the distance between the H374 side chain and the C3 hydroxyl group of cholesterol over the course of equilibration. (B) The same analysis of an H374A mutant. While the wild-type apo Hog model shows stable H-bonding and polar interactions between the hydroxyl group of cholesterol, the alanine residue of an H374A mutant forms no stable contacts.
(TIFF)

S4 Fig The cholesterol tail lies near an essential portion of the hSHH SRR loop. (A,B) A 10 Å sphere from binding site residues suggested by crosslinking (site 1, red) lies at the back of the Hint-SRR interface in the wild type and ΔP392-A423 apo Hog models. (C,D) The MD-optimized model of the wild-type cholesterol-Hog complex and a ΔP392-A423 mutant show that C25 of cholesterol is within 10 Å of this site in both proteins.
(TIFF)

S5 Fig Residues at the Hint-SRR interface shape the cholesterol-Hog complex. (A) A water-mediated H-bond between the L299 backbone carbonyl and the C3-OH group of cholesterol may constrain the position of cholesterol in a stabilized cholesterol-Hog complex. (B) An F380A mutant loses interactions with cholesterol after a 400 ns MD equilibration in the cholesterol-Hog complex. (C) Cholesterol recedes from the binding site of the cholesterol-Hog complex in a Y364A mutant during a 400 ns MD equilibration.
(TIFF)

S6 Fig The tetrahedral adduct forms a stable hydrogen bond. Top: A 400 ns snapshot of a ~1.7 Å H-bond between the G197 oxyanion and the neutral side chain of D243 formed during 400 ns MD simulations. Bottom: A plot of the distance between the oxygen atom at G197 and the D243 carboxylic acid.
(TIFF)

S7 Fig Raw images of gels/blots for Figs 3B and 6B. (TIFF)

S8 Fig Synthesis of 25-diazirinyl-27-norcholest-5-ene-3β-ol (photocholesterol). (TIFF)

S1 Table Plasmid and primer sequences. (PDF)

S1 File Photocholesterol crosslinking/mass spectrometry data. Spectra and assignments for each product ion, modifications, and differences between experimental and theoretical masses.
(PDF)

S2 File Apo hSHH Hog model. (PDB)

S3 File hSHH cholesterol-Hog complex. (PDB)

S4 File hSHH Hog-cholesterol adduct. (PDB)

S5 File (PDF)

S1 Movie Equilibration of “flipped” cholesterol in the binding pocket of the docked model. (MP4)

S2 Movie Migration of cholesterol from the cholesterol-Hog complex to a position for a Hog-cholesterol adduct. (MP4)
