# Supplementary figures and images for: Hedgehog proteins create a dynamic cholesterol interface
Source: PLoS One. 2021 Feb 25;16(2):e0246814. doi: 10.1371/journal.pone.0246814 (PMC7906309; doi:10.1371/journal.pone.0246814)

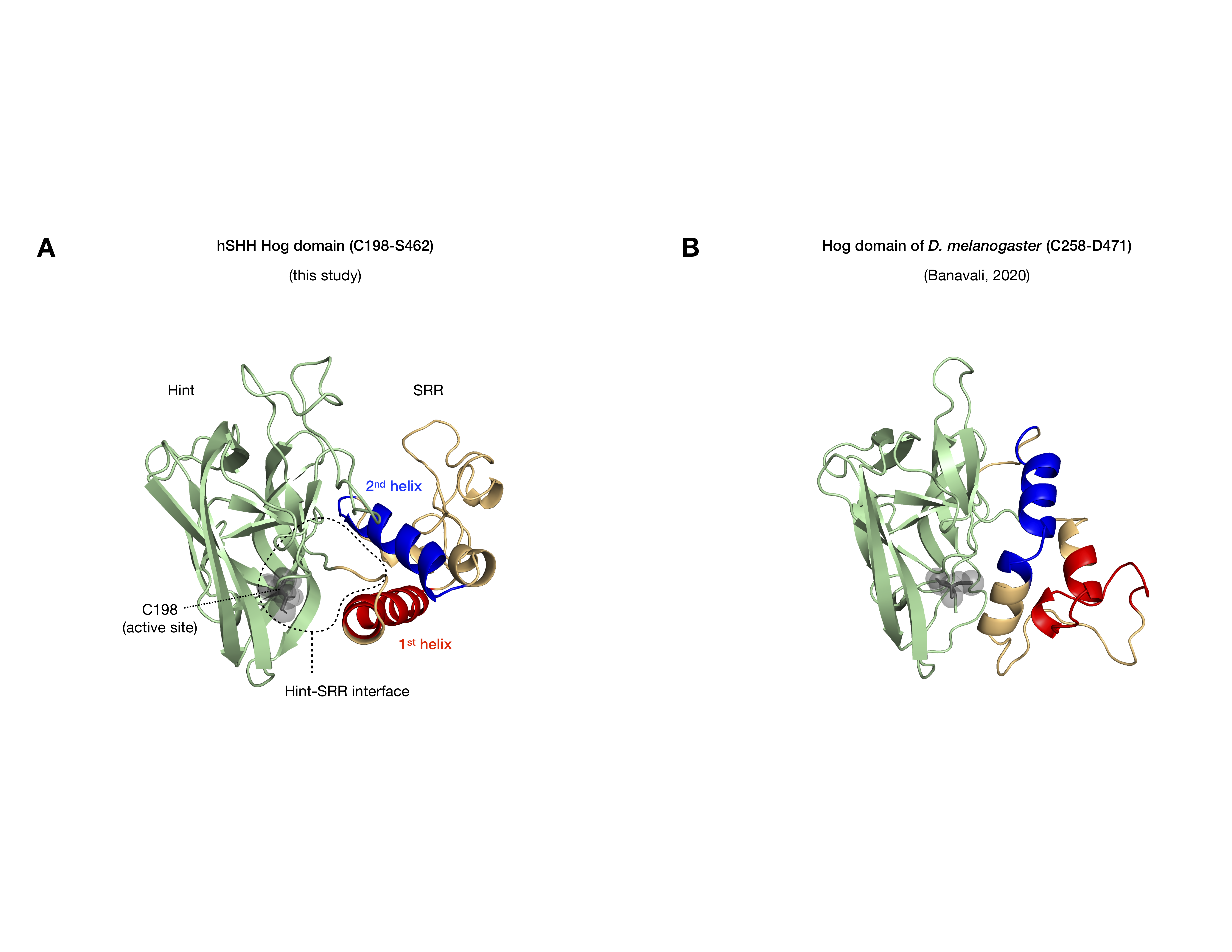

Supplement: S1 Fig — (A) Highest-scoring comparative/ab initio model from the Robetta server, using the crystal structure of D. melanogaster (PDB 1AT0) as a template for the hSHH Hint fold (residues 198–365) and ab initio modeling of the hSHH SRR (residues 366–462). (B) A model of the Hog domain from D. melanogaster Hh (C158-D471), from ref 32. In both models, the 1st SRR helix in a helix-loop-helix motif is colored red; the 2nd SRR helix is colored blue, the SRR loop residues are orange, and the Hint fold is green. Atoms in the active site cysteine residue (C198 in hSHH; C258 in D. melanogaster Hh) are represented as grey spheres. (TIFF) [file pone.0246814.s001.tiff]

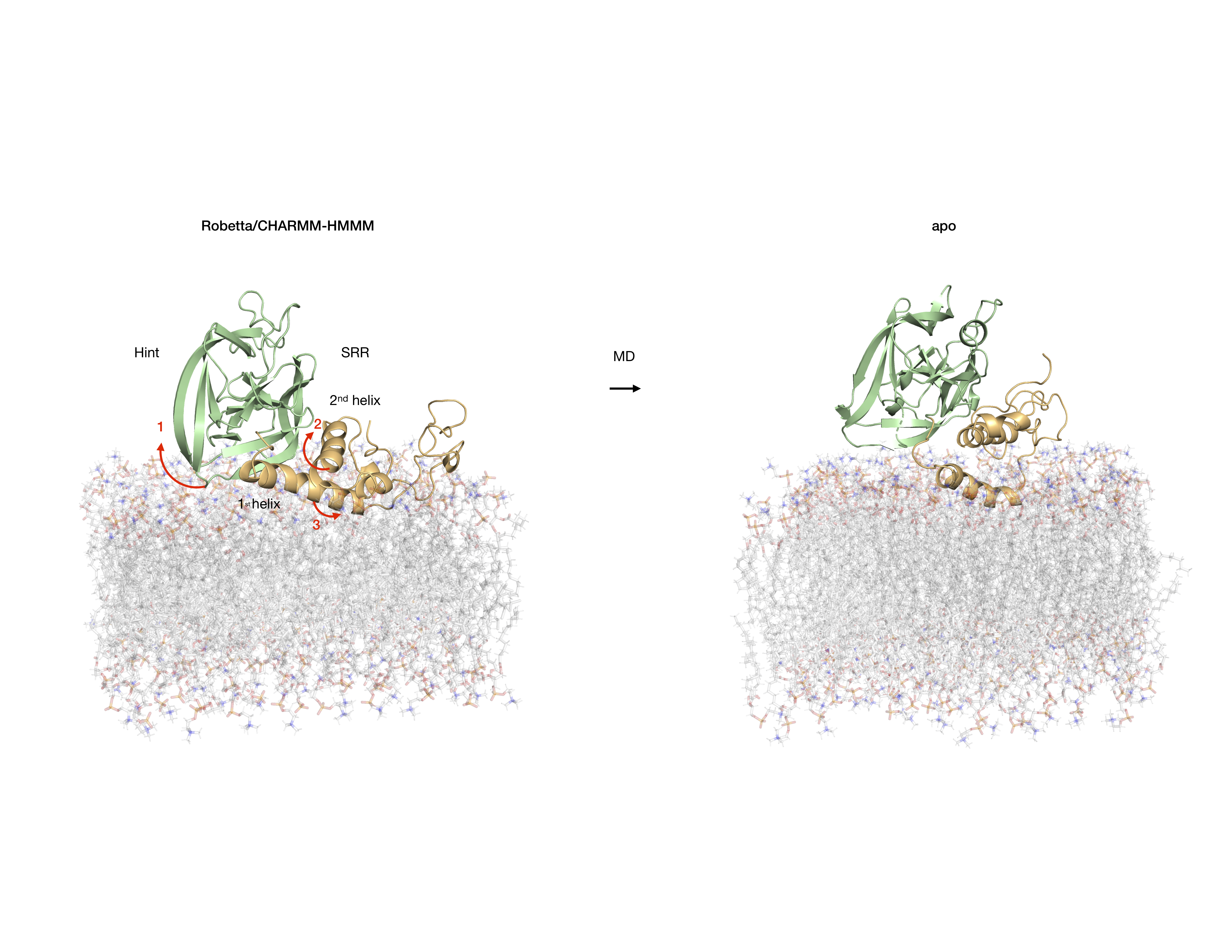

Supplement: S2 Fig — Left: Top-scoring hSHH Hog model from Robetta docked at the membrane using the CHARMM-HMMM server. Right: Model after MD simulations (for details, see Materials and Methods). Red arrows highlight changes during MD simulations. The Hint fold migrates approximately 10 Å from the membrane (1), the 2nd SRR helix rotates approximately 60° to assume an antiparallel arrangement with the 1st SRR helix (2), and the two helices of the SRR separate from a closest Cα-to-Cα distance of 6.2 Å to a distance of 8.1 Å (3). (TIFF) [file pone.0246814.s002.tiff]

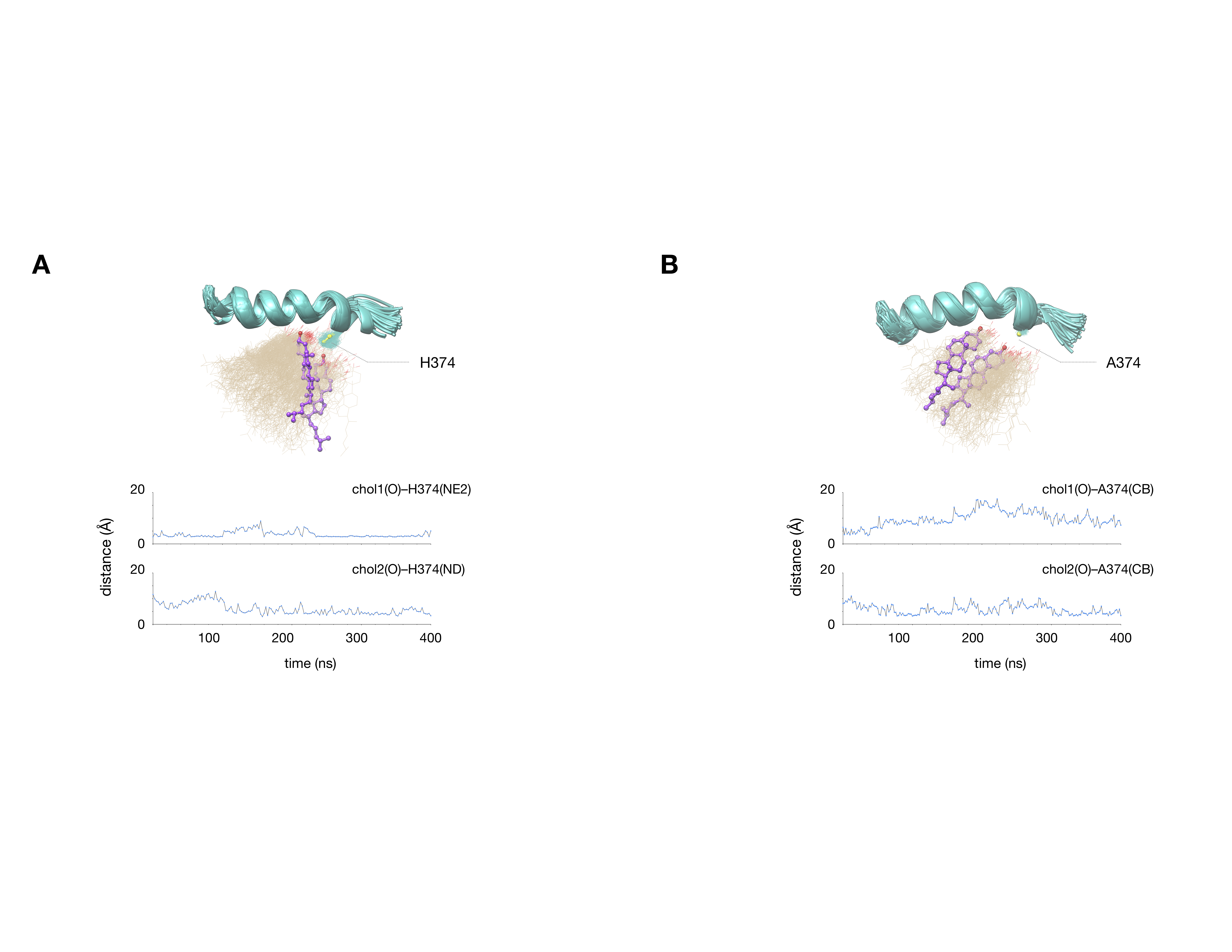

Supplement: S3 Fig — (A) Top: Superposition of 1st SRR helix structures from 400 ns MD equilibration of the wild type apo Hog model. Bottom: Plot of the distance between the H374 side chain and the C3 hydroxyl group of cholesterol over the course of equilibration. (B) The same analysis of an H374A mutant. While the wild-type apo Hog model shows stable H-bonding and polar interactions between the hydroxyl group of cholesterol, the alanine residue of an H374A mutant forms no stable contacts. (TIFF) [file pone.0246814.s003.tiff]

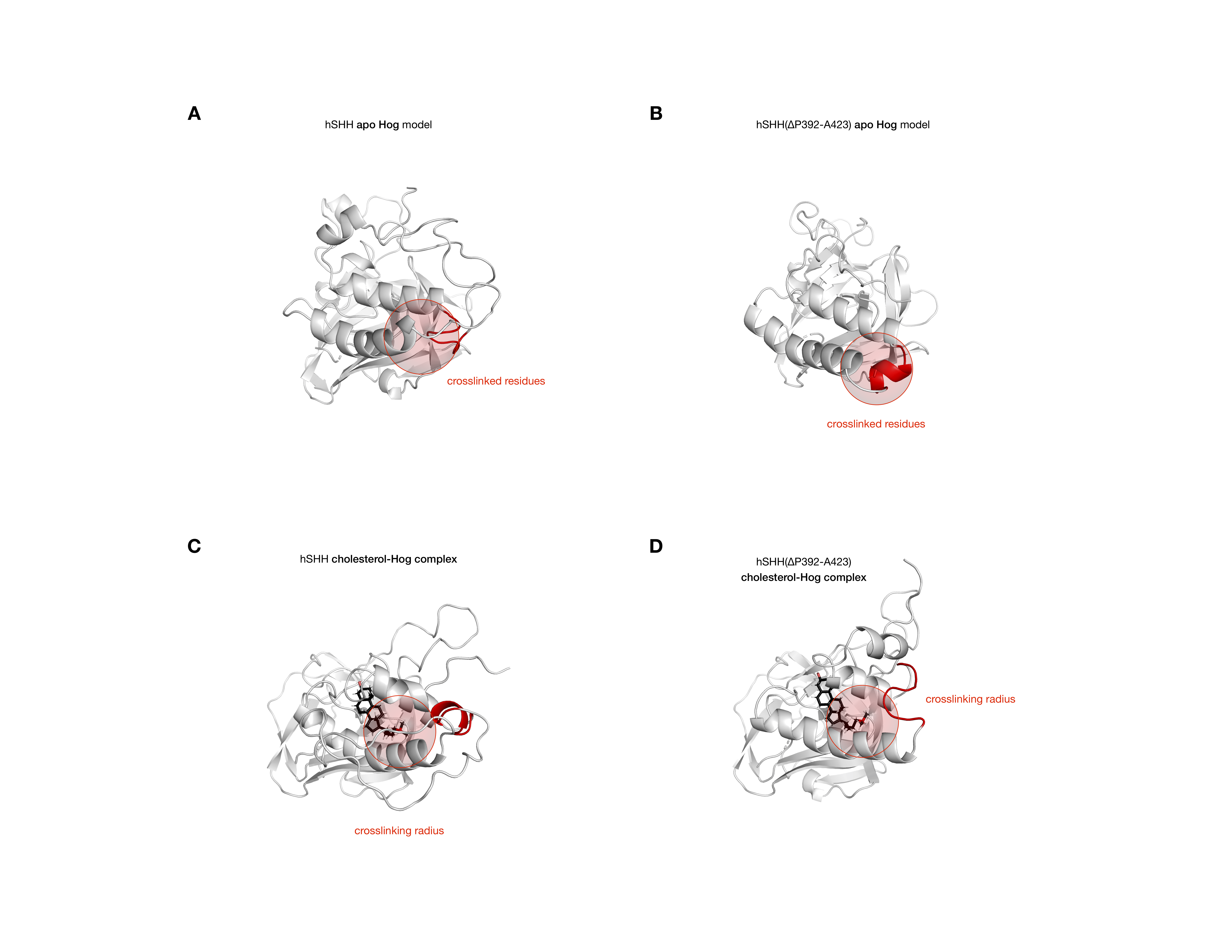

Supplement: S4 Fig — (A,B) A 10 Å sphere from binding site residues suggested by crosslinking (site 1, red) lies at the back of the Hint-SRR interface in the wild type and ΔP392-A423 apo Hog models. (C,D) The MD-optimized model of the wild-type cholesterol-Hog complex and a ΔP392-A423 mutant show that C25 of cholesterol is within 10 Å of this site in both proteins. (TIFF) [file pone.0246814.s004.tiff]

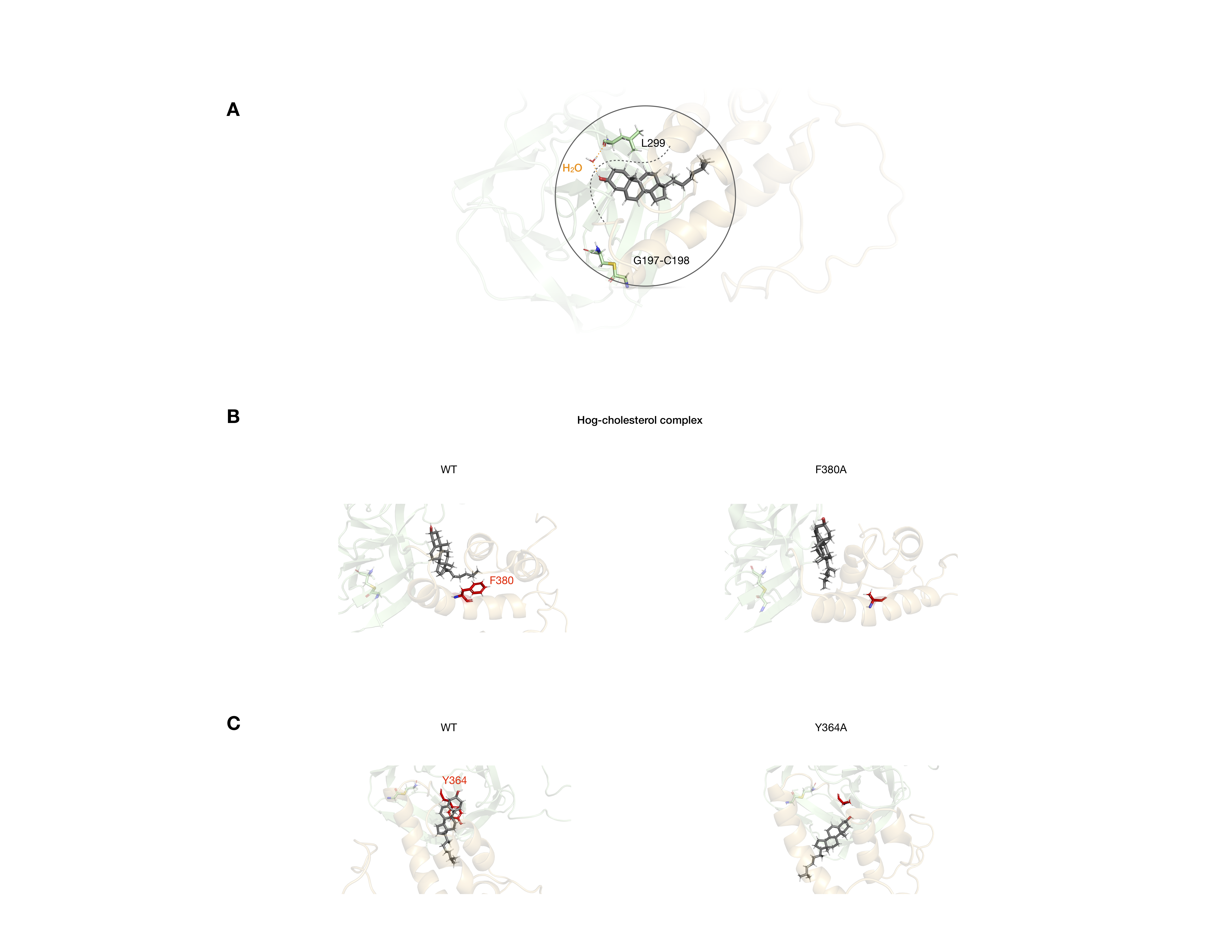

Supplement: S5 Fig — (A) A water-mediated H-bond between the L299 backbone carbonyl and the C3-OH group of cholesterol may constrain the position of cholesterol in a stabilized cholesterol-Hog complex. (B) An F380A mutant loses interactions with cholesterol after a 400 ns MD equilibration in the cholesterol-Hog complex. (C) Cholesterol recedes from the binding site of the cholesterol-Hog complex in a Y364A mutant during a 400 ns MD equilibration. (TIFF) [file pone.0246814.s005.tiff]

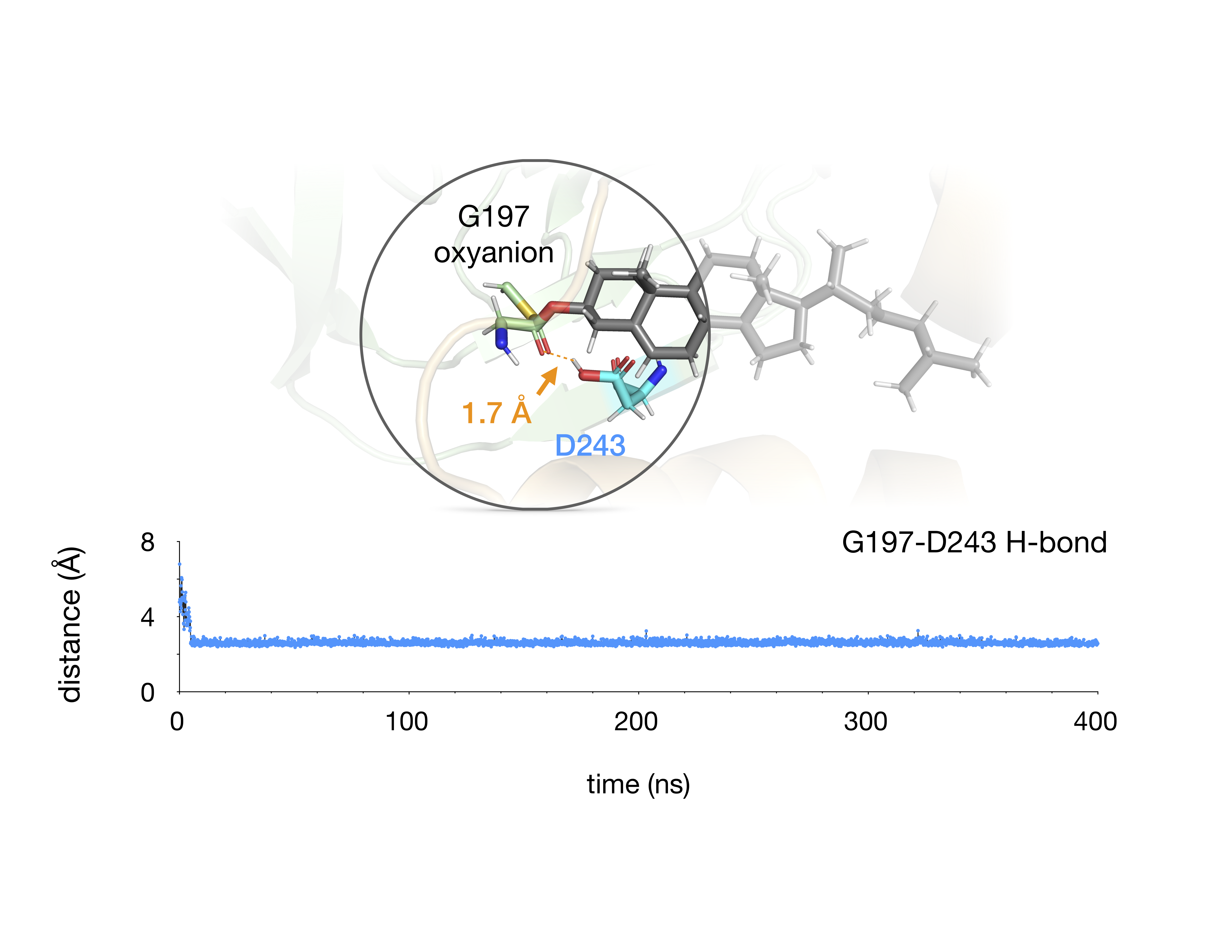

Supplement: S6 Fig — Top: A 400 ns snapshot of a ~1.7 Å H-bond between the G197 oxyanion and the neutral side chain of D243 formed during 400 ns MD simulations. Bottom: A plot of the distance between the oxygen atom at G197 and the D243 carboxylic acid. (TIFF) [file pone.0246814.s006.tiff]

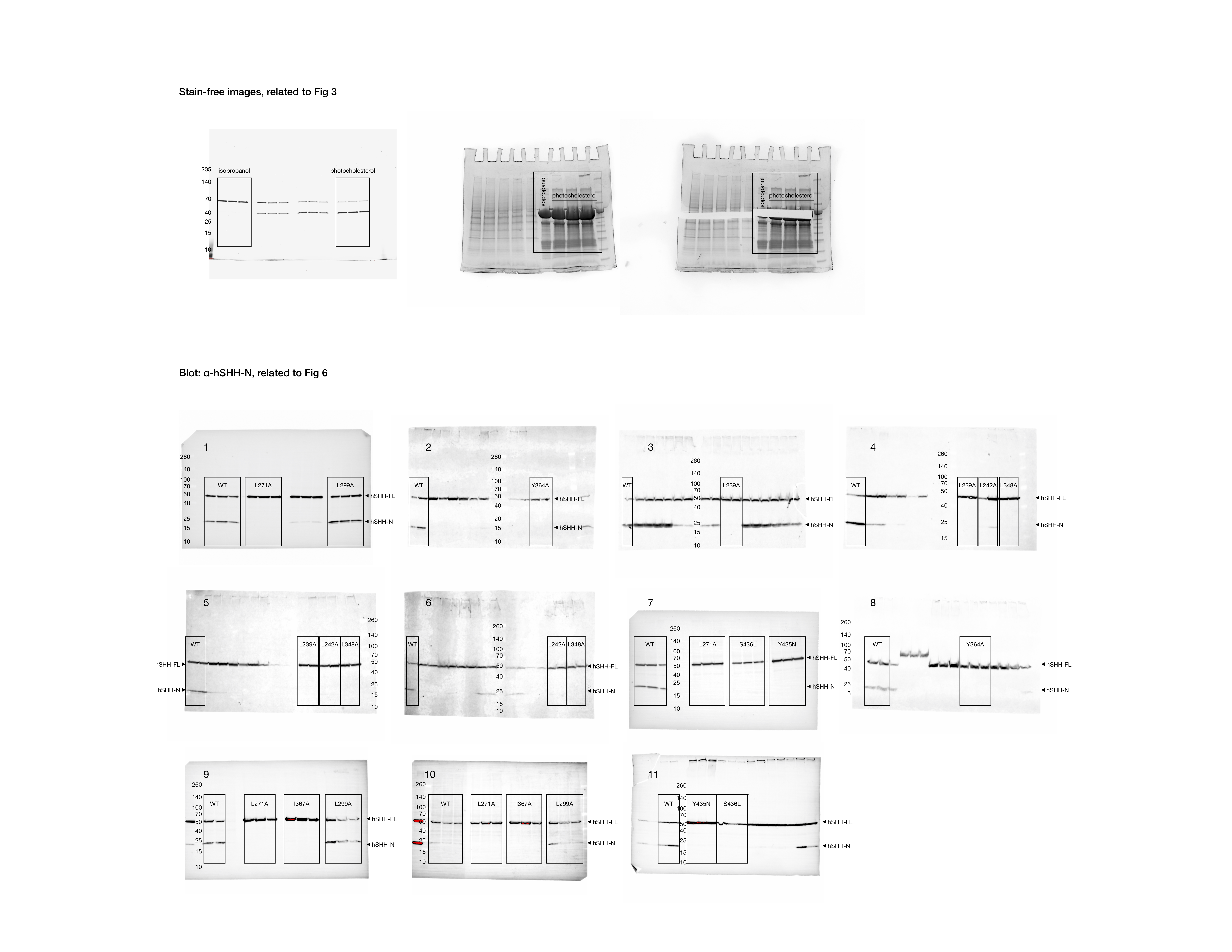

Supplement: S7 Fig — (TIFF) [file pone.0246814.s007.tiff]

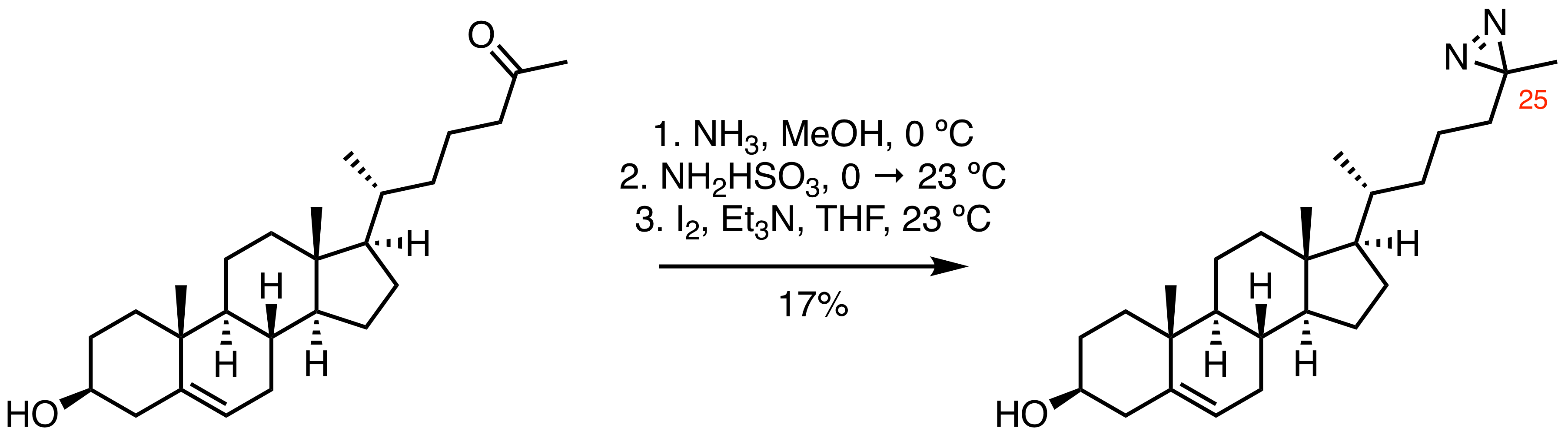

Supplement: S8 Fig — (TIFF) [file pone.0246814.s008.tiff]

**$^1\text{H}$  NMR**

$\text{CDCl}_3$ , 23 °C, 400 MHz

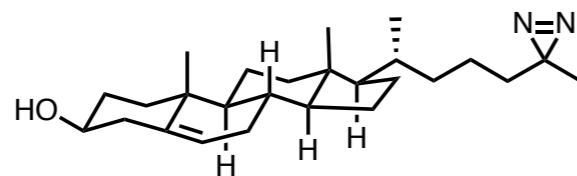

photocholesterol

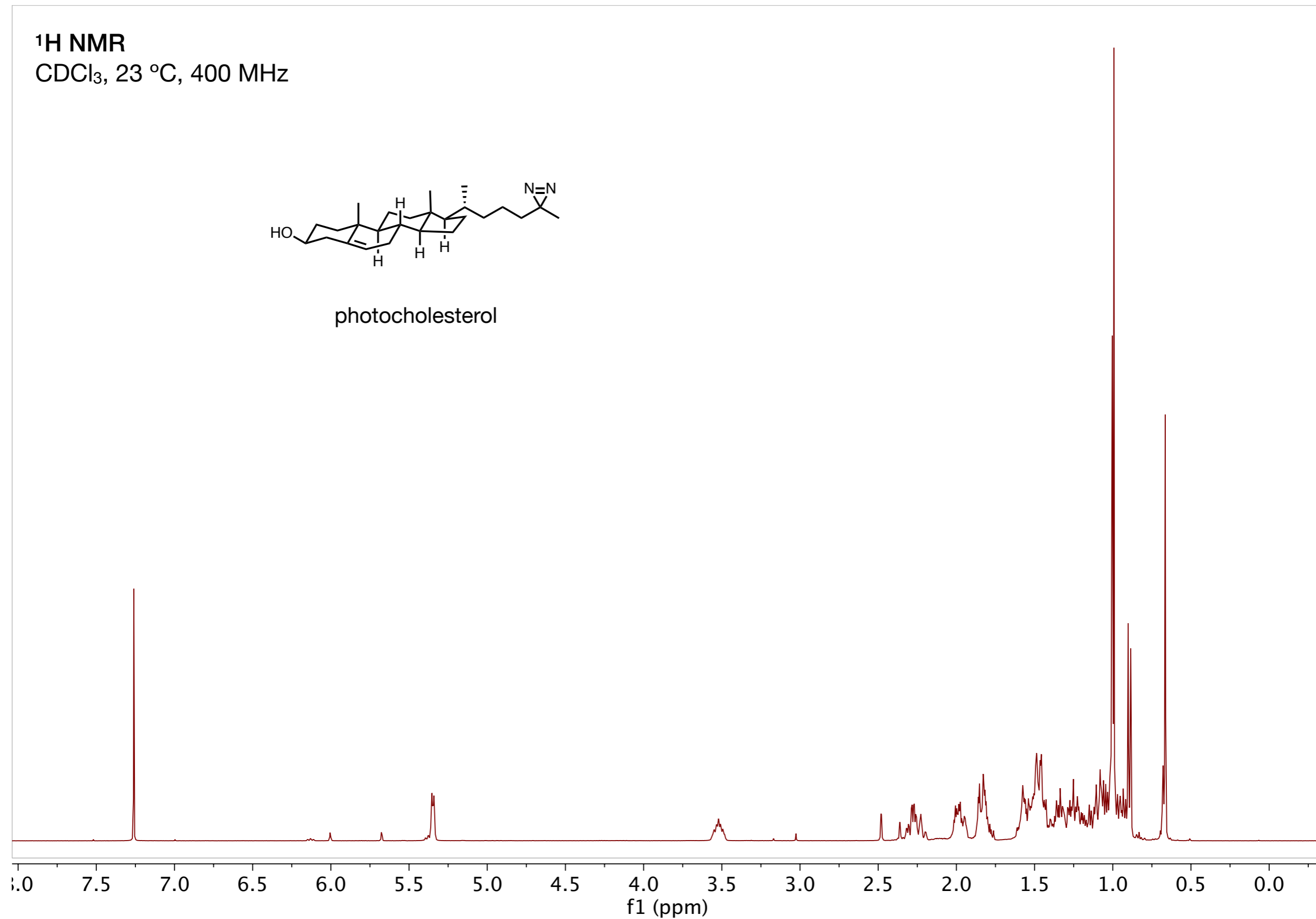

**$^{13}\text{C}$  NMR**

$\text{CDCl}_3$ , 23 °C, 100 MHz

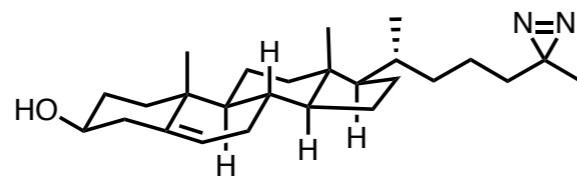

photocholesterol

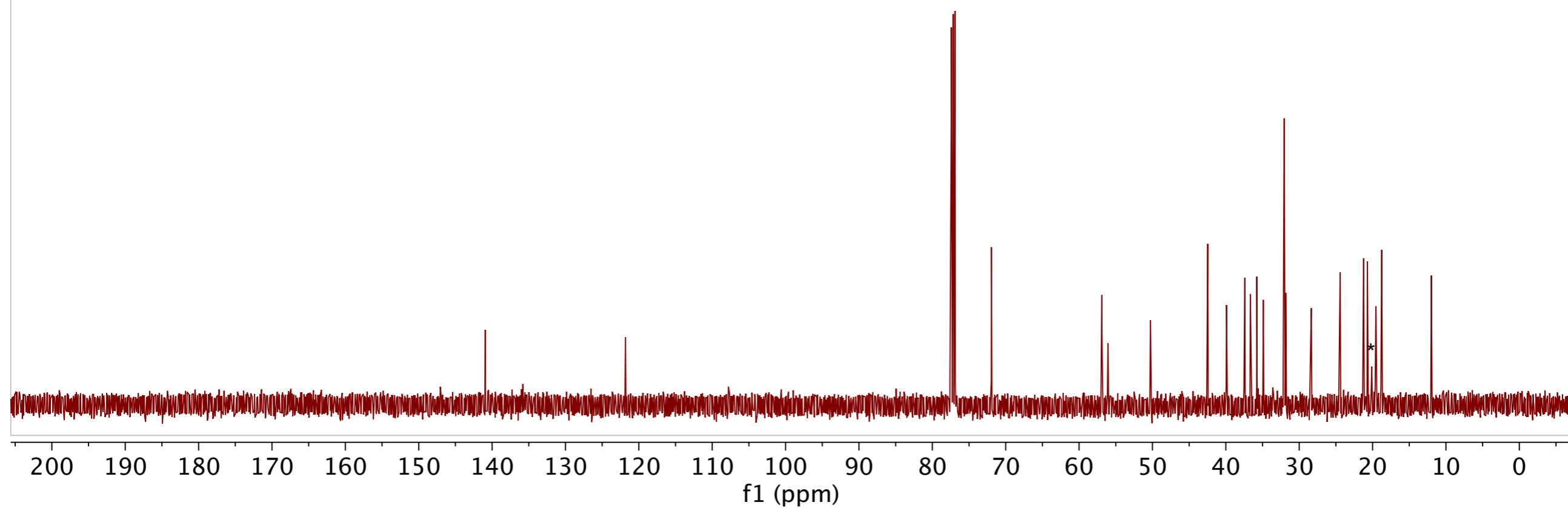

Supplement: S5 File — (PDF) [file pone.0246814.s014.pdf]
