# Supplementary material for: Hedgehog proteins create a dynamic cholesterol interface
Source: PLoS One. 2021 Feb 25;16(2):e0246814. doi: 10.1371/journal.pone.0246814 (PMC7906309; doi:10.1371/journal.pone.0246814)
Supplement: S1 Table — (PDF) [file pone.0246814.s009.pdf]

Table S1. Plasmid and primer sequences.

Plasmid Name	Source
pCMV6-hSHH	Origene RC222175, stop after hSHH(S462)

Mutagenesis primers

Construct	Forward Primer	Reverse Primer
hSHH(F199A)	TCGGGAGGCTGCGCCCCGGGCTCGGC	GCCGAGCCCGGGGCGCAGCCTCCCGA
hSHH(K213A)	GCAGGGCGGCACCGCGCTGGTGAAGGAC	GTCCTTCACCAGCGCGGTGCCGCCCTGC
hSHH(D217A)	AGCTGGTGAAGGCCCTGAGCCCCGG	CCGGGGCTCAGGGCCTTCACCAGCT
hSHH(D222A)	GAGCCCCGGGGCCCGCGTGCTGG	CCAGCACGCGGGCCCCGGGGCTC
hSHH(D245A)	CCTGGACCGCGCCGACGGCGCCA	TGGCGCCGTCGGCGCGGTCCAGG
hSHH(S236A)	GGCCGGCTGCTCTACGCCGACTTCCTCACTTT	AAAGTGAGGAAGTCGGCGTAGAGCAGCCGGCC
hSHH(D237A)	GGCTGCTCTACAGCGCCTTCCTCACTTTCCT	AGGAAAGTGAGGAAGGCGCTGTAGAGCAGCC
hSHH(L239A)	GCTCTACAGCGACTTCGCCACTTTCCTGGACCGC	GCGGTCCAGGAAAGTGGCGAAGTCGCTGTAGAGC
hSHH(F241A)	CTCTACAGCGACTTCCTCACTGCCCTGGACCGCG	CGCGGTCCAGGGCAGTGAGGAAGTCGCTGTAGAG
hSHH(L242A)	AGCGACTTCCTCACTTTCGCGGACCGCGACGA	TCGTCGCGGTCCGCGAAAGTGAGGAAGTCGCT
hSHH(R244A)	CACTTTCCTGGACGCCGACGACGGCGCC	GGCGCCGTCGTCGGCGTCCAGGAAAGTG
hSHH(K249A)	GCGACGACGGCGCCGCGAAGGTCTTCTACG	CGTAGAAGACCTTCGCGGCGCCGTCGTCGC
hSHH(K250A)	CGACGACGGCGCCAAGGCGGTCTTCTACGTGATC	GATCACGTAGAAGACCGCCTTGGCGCCGTCGTCG
hSHH(F252A)	CGGCGCCAAGAAGGTCGCCTACGTGATCGAGACG	CGTCTCGATCACGTAGGCGACCTTCTTGGCGCCG
hSHH(L348A)	GCCTACGCGCCGGCCACGGCCCAGGG	CCCTGGGCCGTGGCCGGCGCGTAGGC
hSHH(L348E)	GCCTACGCGCCGGAGACGGCCCAGGGC	GCCCTGGGCCGTCTCCGGCGCGTAGGC
hSHH(F305A)	CCTCGGGCGCTGGCCGCCAGCCGCGT	ACGCGGCTGGCGGCCAGCGCCCGAGG
hSHH(Y345A)	CCGCGGGCGCCGCCGCGCCGCTCA	TGAGCGGCGCGGCGGCGCCCGCGG
hSHH(Y364A)	GCTGGCCTCGTGCGCCGCGGTCATCGAG	CTCGATGACCGCGGCGCACGAGGCCAGC
