# Supplementary material for: Hedgehog proteins create a dynamic cholesterol interface
Source: PLoS One. 2021 Feb 25;16(2):e0246814. doi: 10.1371/journal.pone.0246814 (PMC7906309; doi:10.1371/journal.pone.0246814)

File S1. Photocholesterol crosslinking mass spectrometry data.

Spectra and assignments for each product ion, modifications, and differences between experimental and theoretical masses.

Index

Summary of identified photocholesterol-modified peptides…………………...……………………………..2

Peptide Match Spectra

Site 1……………………………………………………………………………………………..3

Site 1 unmodified……………………………………………………………………...…………11

Site 2……………………………………………………………………………………………12

Site 3……………………………………………………………………………………………15

Summary of identified photocholesterol-modified peptides.

Site	Identified peptide	#PSM (Rank 1 only)	Photo-cholesterol				Modified residues
			(+)			(-)	
			1	2	3		
1	EQLHSSPK	7	■	■	■		H4/L3/E1
2	SDSSISSHVHGCFTP	1			■		P15
2	SDSSISSHVHGCFTPESTALLESGVR	2			■		S6/S7
3	VLTVTPAHLVSVWQPESQK	1	■				(V1,V4,T5)
3	EQMQNFVQLHTDGGAVLTVTPAHLVSVWQPESQK	1			■		N5
3	NLEQMQNFVQLHTDGGAVLTVTPAHLVSVWQPESQK	2	■				V5/Q4

Site	Identified peptide	#PSM	Photo-cholesterol				Modified residues
			(+)			(-)	
			1	2	3		
1	EQLHSSPKVVSSAQQQNGIHWYANALYK	7	■	■	■	■	no

Annotated Peptide Match Spectra

Peptide sequence: EQLHSSPK (presented in Fig 3C)

Modifications: H3-Photocholesteroyl (+370.32360 Da)

Charge	m/z (Da)	Theo. Mass (Da)	Δ Mass (mmu)	MH+ (Da)	RT
+2	648.40210	648.40232	-0.22	1295.79802	57.3671

Fragment Ions*	m/z (Da)	Theo. Mass (Da)	Δ Mass (mmu)
y_1^+- NH_3	130.08589	130.08626	-0.37
y_1^+	147.11212	147.11280	-0.68
y_2^+- NH_3	227.13885	227.13902	-0.17
b_2^+- H_2O	240.09784	240.09843	-0.59
b_2^+- NH_3	241.08067	241.08190	-1.23
y_2^+	244.16504	244.16557	-0.53
b_2^+	258.10815	258.10845	-0.30
y_3^+	331.19656	331.19760	-1.04
b_3^+	371.19141	371.19251	-1.10
y_4^+- H_2O	400.21887	400.21906	-0.19
y_4^+	418.22891	418.22962	-0.71
y_5^{2+}- H_2O	454.30453	454.30442	0.11
y_6^{2+}- H_2O	510.84442	510.84646	-2.04
b_7^+- H_2O	566.36017	566.34428	15.89
y_7^{2+}- H_2O	574.87433	574.87574	-1.41
y_7^{2+}- NH_3	575.37427	575.36775	6.52
b_4^+- H_2O	860.56384	860.56446	-0.62
b_4^+	878.57251	878.57502	-2.51
y_5^+- H_2O	907.59833	907.60157	-3.24
y_5^+- NH_3	908.60400	908.58559	18.41
y_5^+	925.61072	925.61214	-1.42
b_5^+- H_2O	947.59113	947.59649	-5.36
b_5^+- NH_3	948.59100	948.58050	10.50
b_5^+	965.60211	965.60705	-4.94
y_6^+- H_2O	1020.68469	1020.68564	-0.95
y_6^+- NH_3	1021.68805	1021.66965	18.40
b_6^+- H_2O	1034.62561	1034.62851	-2.90
b_6^+- NH_3	1035.62793	1035.61253	15.40
y_6^+	1038.69653	1038.69620	0.33
b_6^+	1052.64111	1052.63908	2.03
b_7^+- H_2O	1131.68311	1131.68128	1.83
b_7^+	1149.68896	1149.69184	-2.88

*b and y ions that contain the photocholesterol modification are marked in red and blue, respectively.

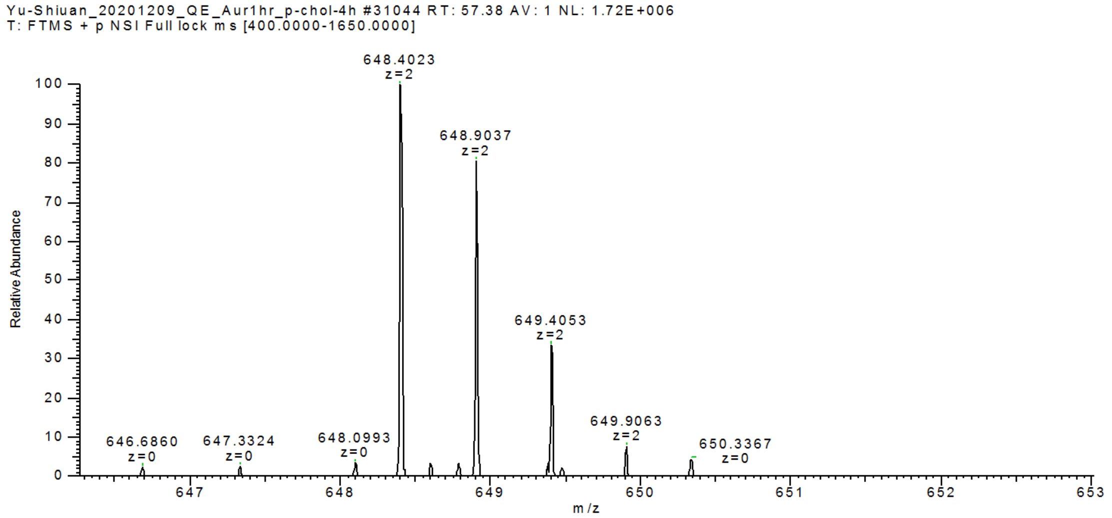

Yu-Shiuan_20201209_QE_Aur1hr_p-chol-4h #31044 RT: 57.38 AV: 1 NL: 1.72E+006
T: FTMS + p NSI Full lock ms [400.0000-1650.0000]
Relative Abundance
m/z
648.4023 z=2
648.9037 z=2
649.4053 z=2
649.9063 z=2
650.3367 z=0
646.6860 z=0
647.3324 z=0
648.0993 z=0

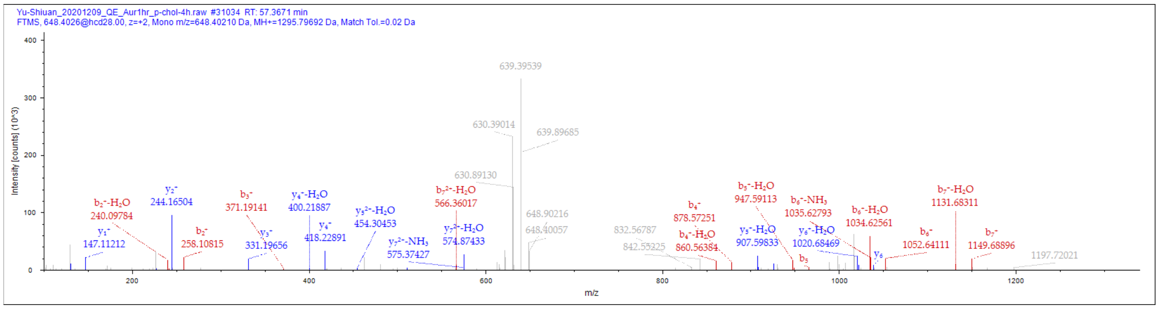

Yu-Shiuan_20201209_QE_Aur1hr_p-chol-4h.raw #31034 RT: 57.3671 min
FTMS, 648.4026@hcd28.00, z=+2, Mono m/z=648.40210 Da, MH+=1295.79692 Da, Match Tol.=0.02 Da
Intensity [counts] (10^3)
m/z

Peptide sequence: EQLHSSPK
Modifications: H3-Photocholesteroyl (+370.32360 Da)

Charge	m/z (Da)	Theo. Mass (Da)	Δ Mass (mmu)	MH+ (Da)	RT
**+3**	432.60373	432.60397	-0.25	1295.79663	57.6313

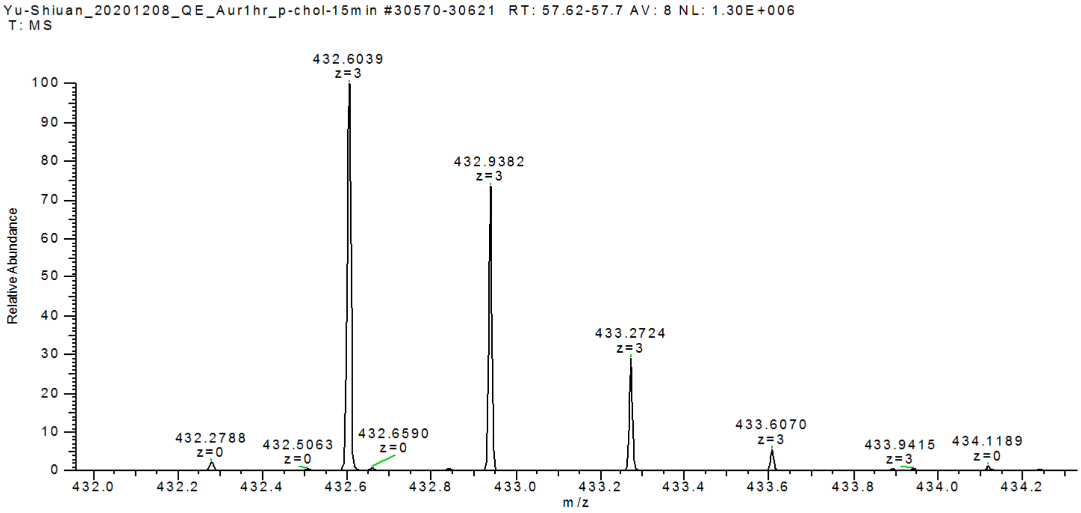

Peptide sequence: EQLHSSPK

Modifications: E1-Photocholesteroyl (+370.32360 Da)

Charge	m/z (Da)	Theo. Mass (Da)	Δ Mass (mmu)	MH+ (Da)	RT
**+3**	432.60410	432.60397	+0.12	1295.79773	61.2433

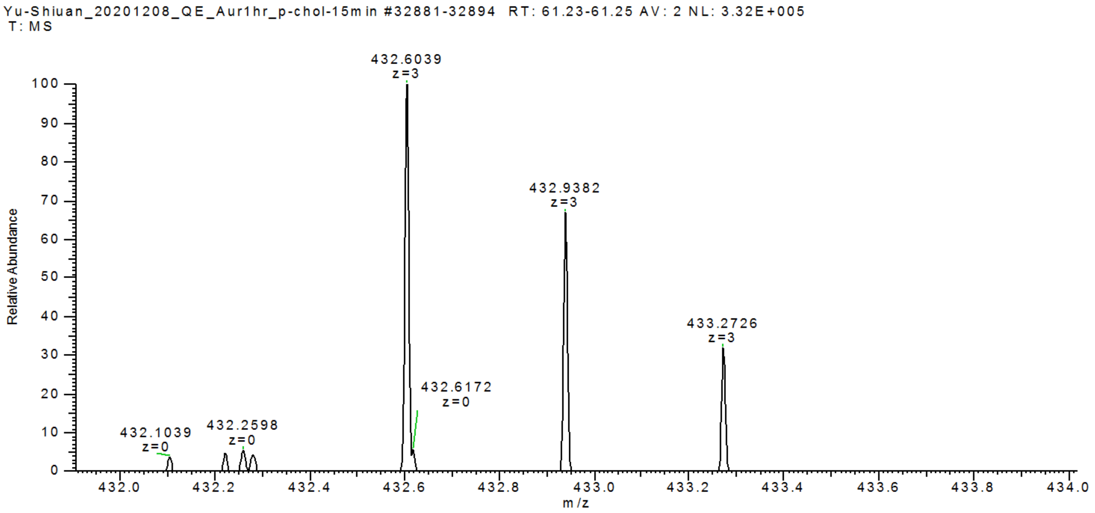

Peptide sequence: EQLHSSPK

Modifications: L3-Photocholesteroyl (+370.32360 Da)

Charge	m/z (Da)	Theo. Mass (Da)	Δ Mass (mmu)	MH+ (Da)	RT
**+2**	648.40308	648.40232	+0.75	1295.79888	57.6276

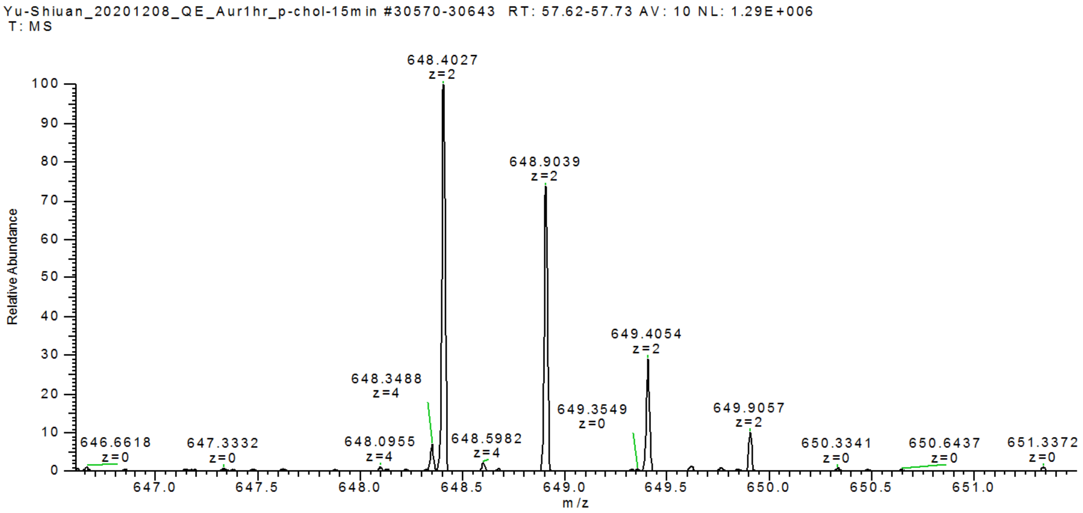

Peptide sequence: EQLHSSPK

Modifications: H4-Photocholesteroyl (+370.32360 Da)

Charge	m/z (Da)	Theo. Mass (Da)	Δ Mass (mmu)	MH+ (Da)	RT
**+3**	432.60345	432.60397	-0.52	1295.79581	55.1098

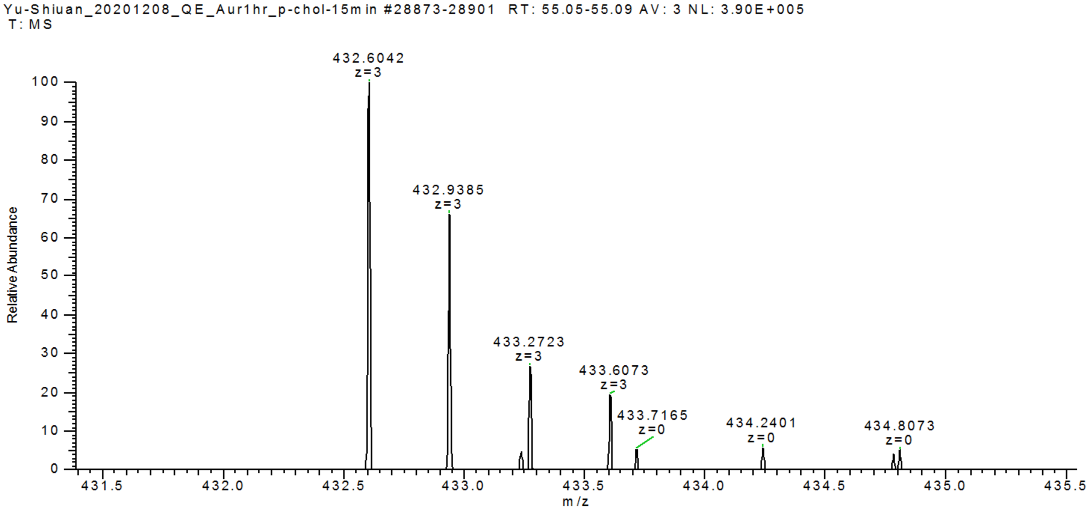

Peptide sequence: EQLHSSPK

Modifications: L3-Photocholesteroyl (+370.32360 Da)

Charge	m/z (Da)	Theo. Mass (Da)	Δ Mass (mmu)	MH+ (Da)	RT
**+2**	648.40332	648.40232	+1.00	1295.79936	55.0835

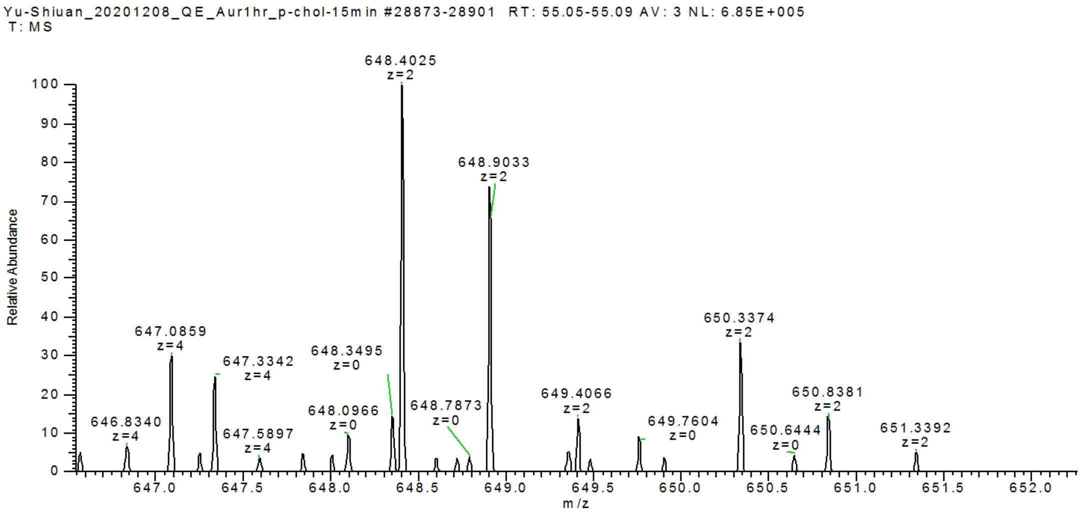

Peptide sequence: EQLHSSPK

Modifications: L3-Photocholesteroyl (+370.32360 Da)

Charge	m/z (Da)	Theo. Mass (Da)	Δ Mass (mmu)	MH+ (Da)	RT
**+2**	648.40265	648.40232	+0.33	1295.79802	56.1154

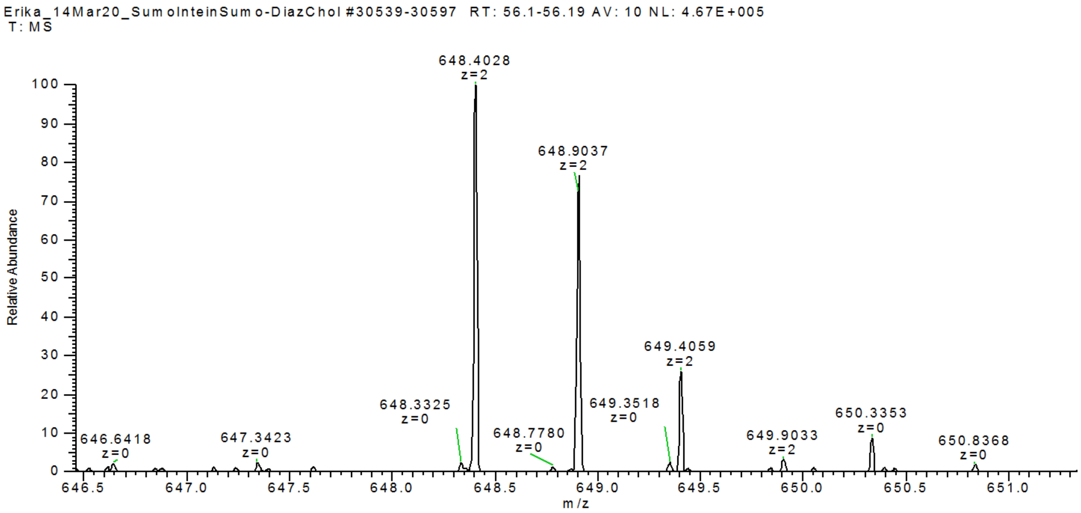

Peptide sequence: SDSSISSHVHGCFTPESTALLESGVR

Modifications: S7-Photocholesteroyl (+370.32360 Da), C12-Carbamidomethyl (+57.02416 Da)

Charge	m/z (Da)	Theo. Mass (Da)	Δ Mass (mmu)	MH+ (Da)	RT
**+4**	783.40735	: 783.40869	-1.35	3130.60756	46.9642

MS1: monoisotopic peak only

Peptide sequence: VLTVTPAHLVSVWQPESQK

Modifications: V1, V4, T5-Photocholesteroyl (+370.32360 Da)

Charge	m/z (Da)	Theo. Mass (Da)	Δ Mass (mmu)	MH+ (Da)	RT
**+3**	1077.37585	1077.38000	-4.14	3230.11301	51.8983

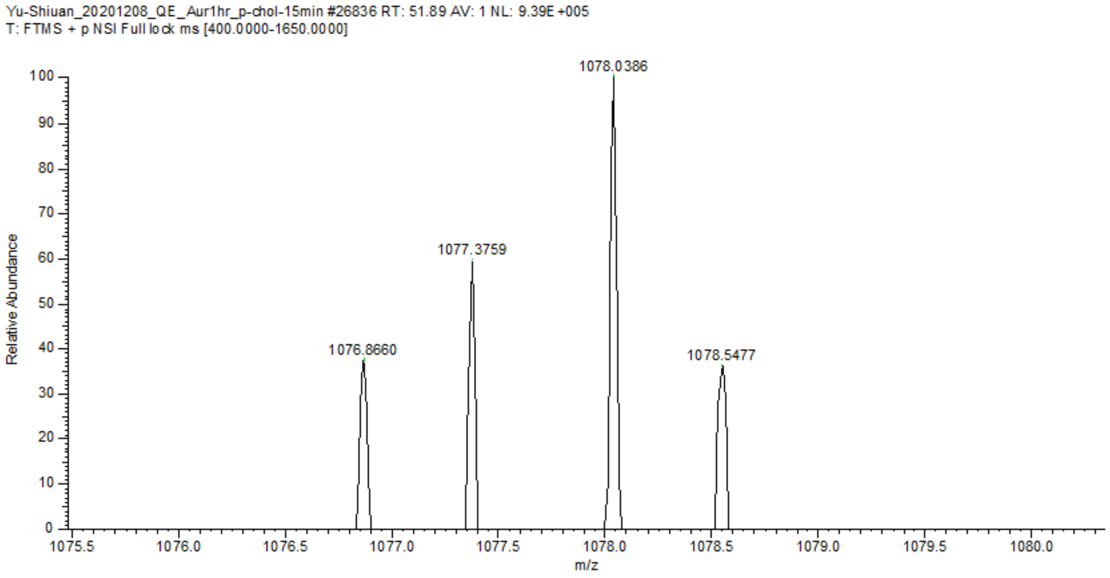

Peptide sequence: EQMQNFVQLHTDGGAVLTVTPAHLVSVWQPESQK

Modifications: N5-Photocholesteroyl (+370.32360 Da), M3-Oxidation (+15.99492 Da)

Charge	m/z (Da)	Theo. Mass (Da)	Δ Mass (mmu)	MH+ (Da)	RT
+3	1387.73083	1387.74130	-10.47	4161.17795	55.3158

Fragment Ions	m/z (Da)	Theo. Mass (Da)	Δ Mass (mmu)
y_3^+	362.20313	362.20341	-0.28
y_5^+	588.29736	588.29877	-1.41
y_7^+	902.43762	902.43666	+0.96
y_9^+	1088.54065	1088.53710	+3.55

MS1: monoisotopic peak only

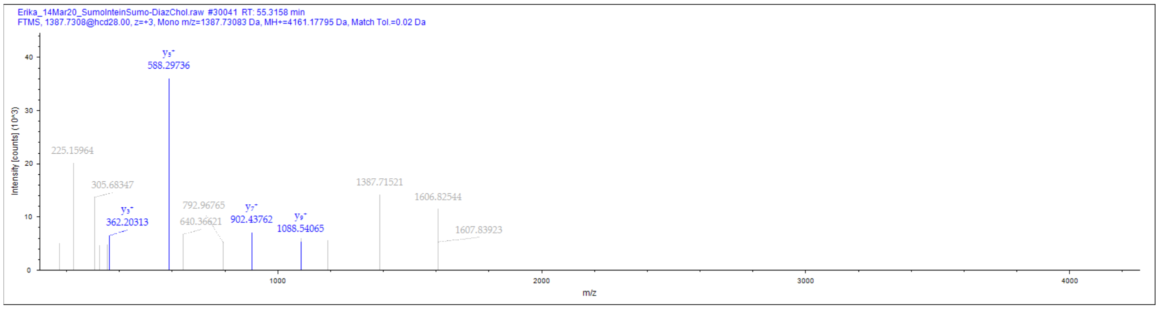

Erika_14Mar20_SumoInteinSumo-DiazChol.raw #30041 RT: 55.3158 min
FTMS, 1387.7308@hcd28.00, z=+3, Mono m/z=1387.73083 Da, MH+=4161.17795 Da, Match Tol.=0.02 Da
Intensity [counts] (10^3)
m/z
225.15964
305.68347
y3+
362.20313
y5+
588.29736
792.96765
640.36621
y7+
902.43762
y9+
1088.54065
1387.71521
1606.82544
1607.83923

Peptide sequence: SDSSISSHVHGCFTP

Modifications: P15-Photocholesteroyl (+370.32360 Da), C12-Carbamidomethyl (+57.02416 Da)

Charge	m/z (Da)	Theo. Mass (Da)	Δ Mass (mmu)	MH+ (Da)	RT
+3	663.34229	648.40232	-2.5	1988.01230	31.5760

Fragment Ions	m/z (Da)	Theo. Mass (Da)	Δ Mass (mmu)
**b_2^{2+}**	102.05539	102.03676	+18.63
**b_4^{2+}**	189.08701	189.06879	+18.22
**b_2^{+}**	203.06616	203.06625	-0.09
**b_3^{+}- H_2O**	272.08792	272.08771	+0.21
**b_3^{+}**	290.09851	290.09828	+0.23
**b_4^{+}- H_2O**	359.11862	359.11974	-1.12
**b_5^{+}- H_2O**	472.20340	472.20380	-0.40
**b_{11}^{2+}**	547.74915	574.74670	+2.45
**b_{12}^{2+}**	627.75665	627.76203	-5.38
**b_{13}^{2+}**	701.29102	701.29623	-5.21
**b_{14}^{2+}- H_2O**	742.80750	742.81479	-7.29
**b_{14}^{2+}**	751.81696	751.82007	-3.11
**b_8^{+}**	801.33582	801.33734	-1.52
**b_{10}^{+}**	1037.46326	1037.46466	-1.40

MS1: monoisotopic peak only

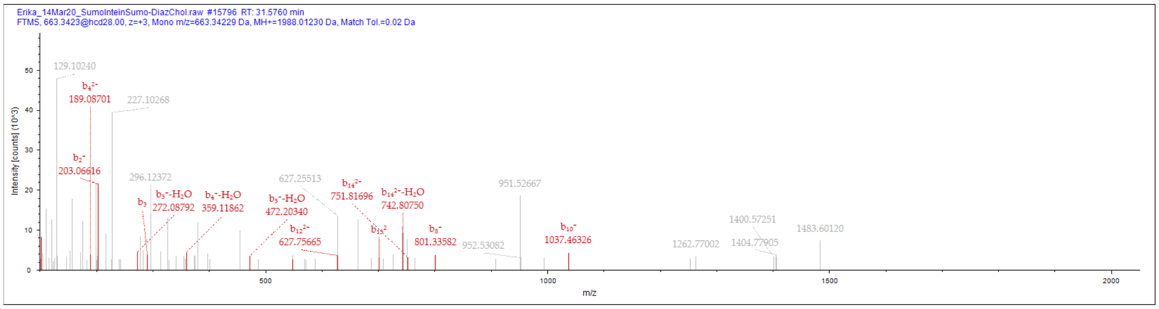

Peptide sequence: NLEQMQNFVQLHTDGGAVLTVTPAHLVSVWQPESQK

Modifications: V5-Photocholesteroyl (+370.32360 Da)

Charge	m/z (Da)	Theo. Mass (Da)	∆ Mass (mmu)	MH+ (Da)	RT
+3	1458.10645	1458.11866	-12.21	4372.30478	56.7616

Fragment Ions	m/z (Da)	Theo. Mass (Da)	∆ Mass (mmu)
**b_2^+**	228.13454	228.13427	+0.27
**b_3^+**	357.17657	357.17686	-0.29
**y_3^+**	362.20404	362.20341	+0.63
**y_5^+- H_2O**	570.28839	570.28820	+0.19
**y_{10}^+- H_2O**	585.30042	585.30111	-0.69
**y_5^+**	588.30011	588.29877	+1.34
**y_6^+- H_2O**	698.34778	698.34678	+1.00
**y_6^+- NH_3**	699.33301	699.33080	+2.21
**y_7^+**	902.43378	902.43666	-2.88
**y_9^+**	1088.54309	1088.53710	+5.99
**y_9^+**	1300.69531	1300.68958	+5.73
**y_{14}^+**	1605.83569	1605.83837	-2.68

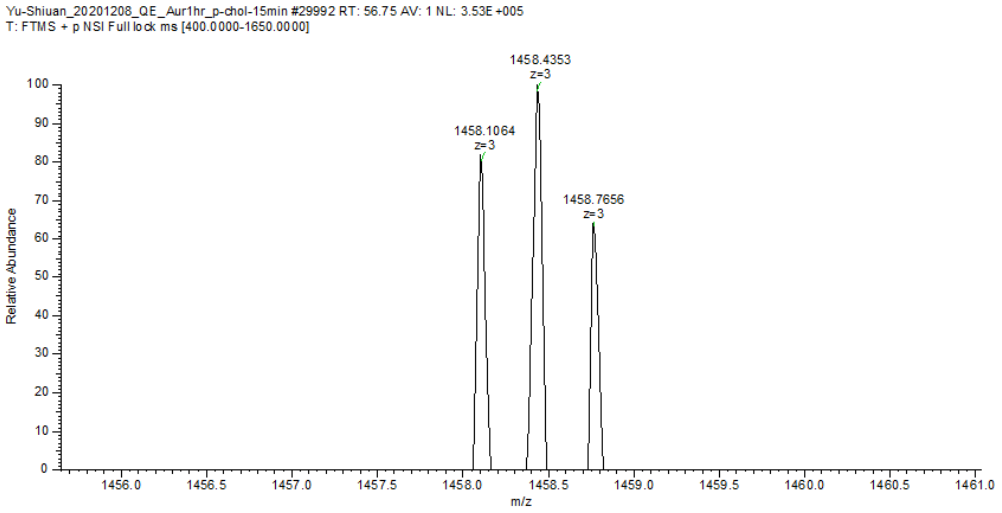

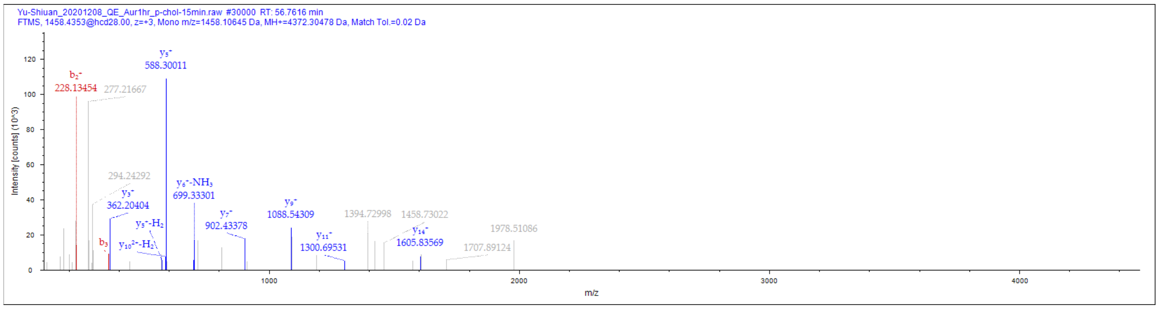

Peptide sequence: NLEQMQNFVQLHTDGGAVLTVTPAHLVSVWQPESQK

Modifications: Q4-Photocholesteroyl (+370.32360 Da), M5-Oxidation (+15.99492 Da)

Charge	m/z (Da)	Theo. Mass (Da)	∆ Mass (mmu)	MH+ (Da)	RT
+5	878.46570	878.47309	-7.39	4388.29938	52.9491

Fragment Ions	m/z (Da)	Theo. Mass (Da)	∆ Mass (mmu)
**y_1^+- NH_3**	130.08606	130.08626	-0.20
**y_1^+**	147.11287	147.11280	+0.07
**y_8^{5+}**	201.12344	201.10684	+16.60
**b_2^+**	228.13374	228.13427	-0.53
**y_2^+- NH_3**	258.14505	258.14483	+0.22
**y_5^{2+}- NH_3**	286.13953	286.13975	-0.22
**b_3^+**	357.17648	357.17686	-0.38
**y_3^+**	362.20328	362.20341	-0.13
**y_{17}^{5+}**	382.20865	382.20625	+2.40
**y_4^+**	491.24811	491.24600	+2.11
**y_5^+**	588.29828	588.29877	-0.49
**y_6^+- NH_3**	699.33270	699.33080	+1.90
**y_6^+**	716.35675	716.35734	-0.59
**y_{14}^{2+}**	803.42438	803.42282	+1.56
**y_7^+**	902.42480	902.43666	-11.86
**y_{17}^{2+}**	953.99738	954.00471	-7.33
**y_9^+**	1088.54016	1088.53710	+3.06
**y_{10}^+**	1187.60828	1187.60551	+2.77
**y_{11}^+**	1300.69446	1300.68958	+4.88

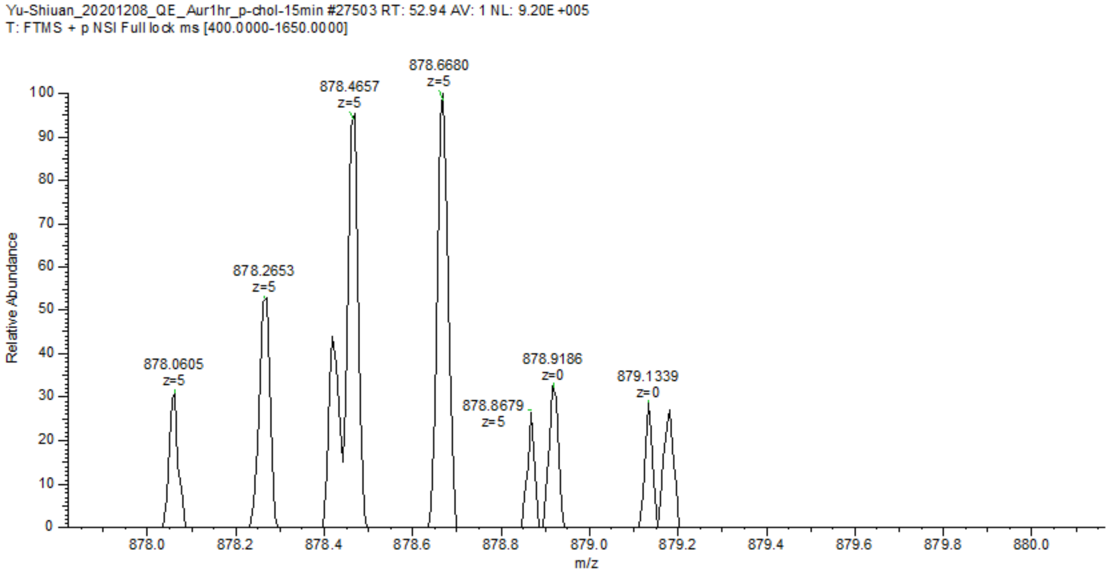

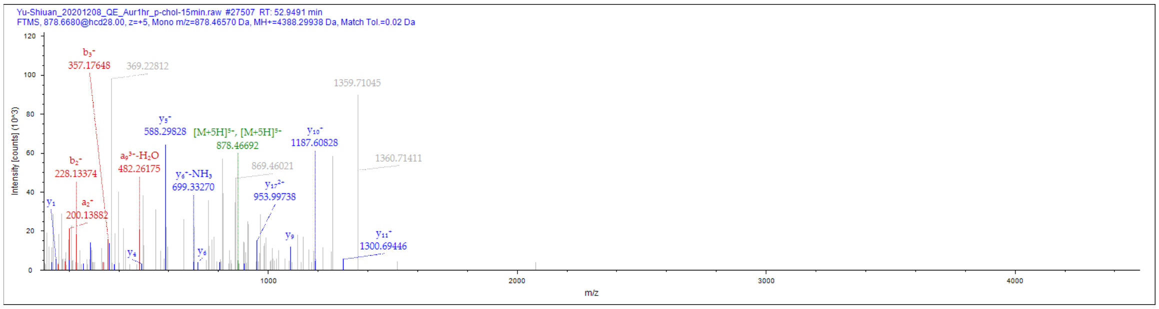
Yu-Shiuan_20201208_QE_Aur1hr_p-chol-15min.raw #27507 RT: 52.9491 min
FTMS, 878.6680@hcd28.00, z=+5, Mono m/z=878.46570 Da, MH+=4388.29938 Da, Match Tol.=0.02 Da
Intensity [counts] (10^3)
m/z
b3+ 357.17648
369.22812
1359.71045
y5+ 588.29828
[M+5H]5+, [M+5H]5+ 878.46692
y10+ 1187.60828
1360.71411
b2+ 228.13374
a9 3+-H2O 482.26175
869.46021
y6+-NH3 699.33270
y17 2+ 953.99738
y1
a2+ 200.13882
y9
y11+ 1300.69446
y4
y6

Peptide sequence: SDSSISSHVHGCFTPESTALLESGVR

Modifications: S6-Photocholesteroyl (+370.32360 Da), C12-Carbamidomethyl (+57.02416 Da)

Charge	m/z (Da)	Theo. Mass (Da)	∆ Mass (mmu)	MH+ (Da)	RT
+4	783.41010	783.40869	+1.4	3130.61855	58.1743

Fragment Ions	m/z (Da)	Theo. Mass (Da)	∆ Mass (mmu)
y_4^+	418.24057	418.24086	-0.29
b_9^{3+}- H_2O	418.24057	418.24445	-3.88
y_5^+	547.28430	547.28345	+0.85
y_6^+	660.36694	660.36752	-0.58
y_7^+	774.45239	773.45158	+0.81

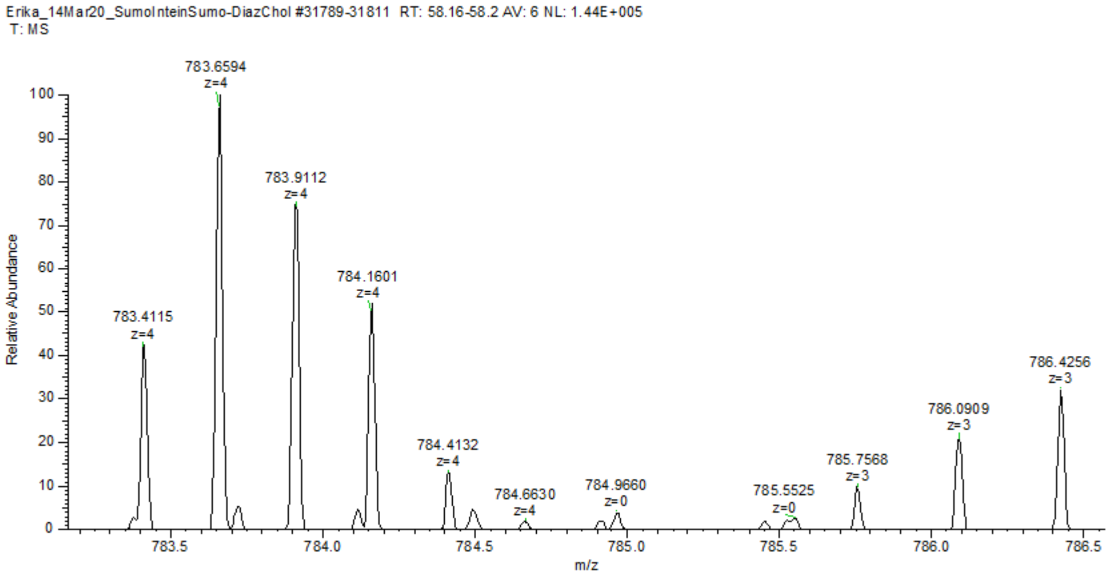

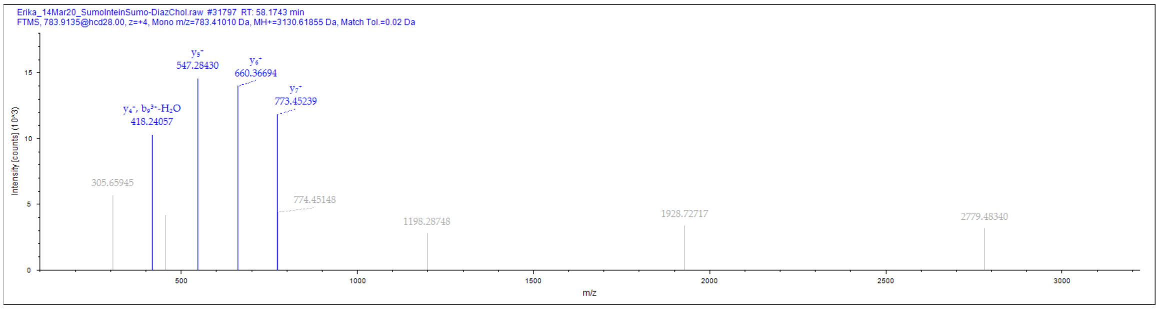

Peptide sequence: <u>SDSSISSHVHGCFTPESTALLESGVR</u>
Modifications: S7-Photocholesteroyl (+370.32360 Da), C12-Carbamidomethyl (+57.02416 Da)

Charge	m/z (Da)	Theo. Mass (Da)	Δ Mass (mmu)	MH+ (Da)	RT
+4	783.40735	: 783.40869	-1.35	3130.60756	46.9642

MS1: monoisotopic peak only

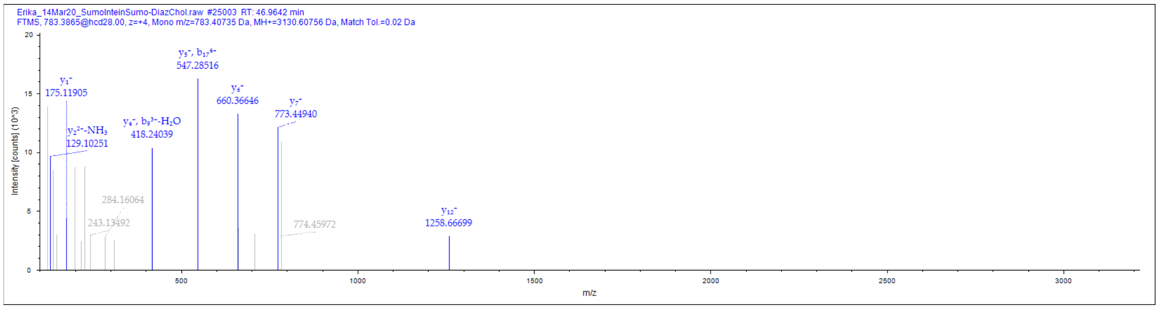

Peptide sequence: VLTVTPAHLVSVWQPESQK

Modifications: V1, V4, T5-Photocholesteroyl (+370.32360 Da)

Charge	m/z (Da)	Theo. Mass (Da)	Δ Mass (mmu)	MH+ (Da)	RT
+3	1077.37585	1077.38000	-4.14	3230.11301	51.8983

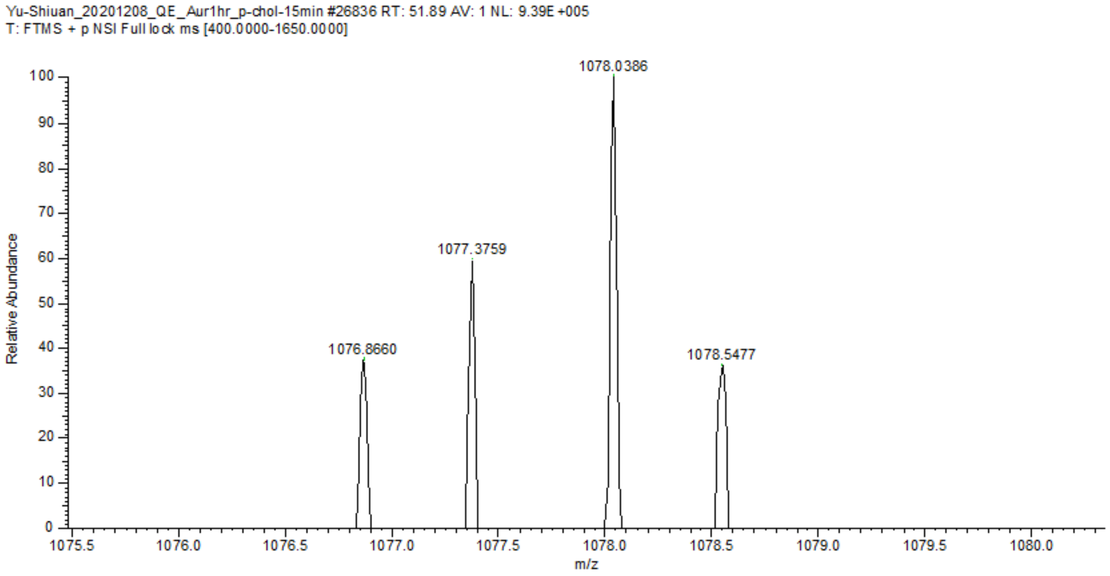

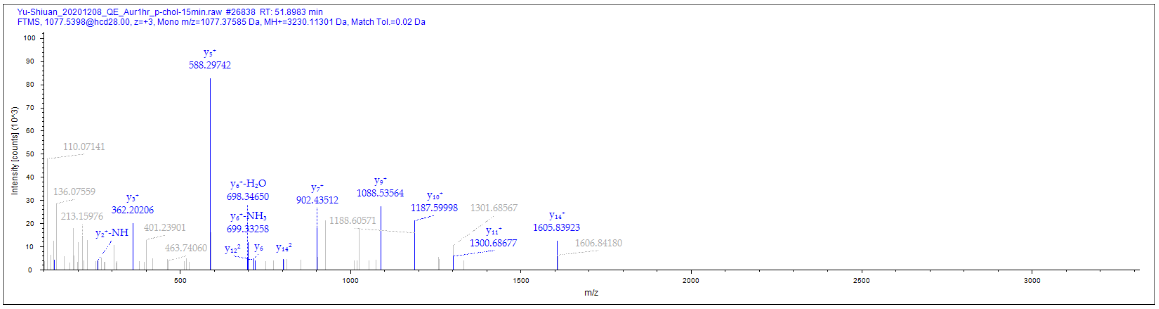

Supplement: S1 File — Spectra and assignments for each product ion, modifications, and differences between experimental and theoretical masses. (PDF) [file pone.0246814.s010.pdf]
